# Structural basis for the allosteric regulation and catalytic mechanism of *Staphylococcus aureus* UMP kinase

**DOI:** 10.3389/fmicb.2025.1733028

**Published:** 2026-01-14

**Authors:** Yan Gao, Zhongliang Zhu, Lianyu Wang, Jiyuan Ke, Liwen Niu

**Affiliations:** 1 MOE Key Laboratory for Membraneless Organelles and Cellular Dynamics, University of Science and Technology of China, Hefei, Anhui, China; 2 Division of Life Sciences and Medicine, School of Life Sciences, University of Science and Technology of China, Hefei, Anhui, China; 3 Hefei National Laboratory for Physical Sciences at the Microscale, University of Science and Technology of China, Hefei, Anhui, China; 4 Institute of Health and Medicine, Hefei Comprehensive National Science Center, Hefei, Anhui, China

**Keywords:** allosteric regulation, antimicrobial target, conformational rearrangement, crystal structure, *Staphylococcus aureus*, UMPK

## Abstract

*Staphylococcus aureus* uridine monophosphate kinase (saUMPK) functions as a hexameric enzyme that catalyzes the reversible reaction: Mg^2+^ ⋅ ATP + UMP ↔ Mg^2+^ ⋅ ADP + UDP. As a key enzyme in pyrimidine metabolism with no detectable homologs in eukaryotes, saUMPK represents an attractive antibacterial target. In this study, we determined crystal structures of saUMPK in complex with various nucleotides, including UMP (3.26 Å), UDP (2.75 Å), GTP (2.88 Å), UTP (2.30 Å), ATP/GTP (2.88 Å), and ATP/UMP (2.57 Å), and performed complementary biochemical assays. Structurally, our analyses revealed several key findings: (1) We captured a previously unobserved apo-like conformation of saUMPK; (2) We identified key residues involved in UMP recognition and revealed the substrate-binding plasticity at the ATP donor site; (3) We uncovered that the allosteric site accommodates different nucleotides through a conserved network of basic residues (R101, R119, R122, K126, and R128). Notably, both the type and number of bound nucleotides cooperatively regulate the final conformational state of the saUMPK hexamer. GTP molecules fully occupy the allosteric sites, stabilizing the open conformation and preserving the global threefold symmetry. In contrast, UTP, ATP, or UDP only partially occupy the allosteric sites, resulting in a loss of this symmetry, while ATP or UDP binding further induces a U-shaped closed conformation of the hexamer. Site-directed mutagenesis identified key residues critical for enzymatic activity. These insights provide a foundation for designing broad-spectrum inhibitors targeting UMP kinase from *Staphylococcus aureus* and related Gram-positive bacteria.

## 1 Introduction

*Staphylococcus aureus* is a widely distributed Gram-positive opportunistic pathogen in nature, characterized by strong environmental adaptability (Jenul and Horswill, 2019; Suthi et al., 2023). This bacterium can invade the host by forming biofilms in the nasal cavity, skin wounds, or on indwelling medical devices, and it can also cause toxin-mediated infections through contaminated food, leading to a wide range of infections and complications in humans, and occasionally death (Ogonowska et al., 2020; Cheung et al., 2021; Liu et al., 2025). In cases of co-infection with COVID-19, the risk of patient mortality is significantly increased (Gu et al., 2025). In addition, this bacterium can be transmitted to humans through agricultural and aquacultural carriers such as pigs, cattle, and seafood, posing a cross-species health threat (Krueger et al., 2025). The growing problem of antibiotic resistance, exemplified by methicillin-resistant *Staphylococcus aureus* (MRSA), has intensified the global public health burden and underscores the urgent need for new antibacterial agents (Lowy, 1998; Abreu et al., 2012; Cheung et al., 2021; Suthi et al., 2023).

The bacterial uridine monophosphate kinase (UMPK) is a key enzyme that catalyzes the conversion of UMP to UDP by transferring the γ-phosphate group from ATP. It catalyzes a key step in UTP biosynthesis (Briozzo et al., 2005; Marco-Marín et al., 2005; Chu et al., 2012). Through its regulation of nucleotide metabolism, UMPK contributes to bacterial survival adaptability, such as cell wall and capsule synthesis and maintenance of osmotic pressure, as well as pathogenic processes, including resistance to host phagocytosis and adhesion/colonization (Grundling and Schneewind, 2007; Swarupa et al., 2017; Paton and Trappetti, 2019; Cheung et al., 2021; Yu et al., 2024). UMPK is encoded by the *pyrH* gene, which is fully conserved across all *Staphylococcus aureus* strains, including MRSA and the highly virulent MW2 strain (Hari Prasad et al., 2012). As one of the key proteins preferentially expressed during host invasion, deletion of *pyrH* leads to bacterial lethality (Lee et al., 2007). Notably, bacterial UMPK shares no homology with human UMP/CMP kinase. Therefore, it is considered a relatively safe and broad-spectrum antibacterial target (Doig et al., 2013).

*Staphylococcus aureus* UMPK (saUMPK) belongs to bacterial UMPKs, and originates from a distinct subclass within the nucleoside monophosphate kinase (NMPK) superfamily. All bacterial UMPKs possess three nucleotide-binding sites—an allosteric site, an ATP donor site, and a UMP acceptor site—and typically assemble into homohexameric complexes. The spatial separation of the allosteric and catalytic sites enables allosteric regulation of enzyme activity by different small nucleotides (Evrin et al., 2007; Chu et al., 2012). In Gram-negative bacteria, such as *Escherichia coli*, the allosteric site specifically binds GTP, while UTP acts as a competitive inhibitor at the active site (Briozzo et al., 2005; Meyer et al., 2008). In contrast, Gram-positive bacteria including *Staphylococcus aureus* can accommodate different small nucleotides in the allosteric site and exhibit distinct responses to GTP, UTP, and ATP (Evrin et al., 2007; Meier et al., 2008; Labesse et al., 2011; Walter et al., 2022). Although archaeal UMPKs share a hexameric structure with their bacterial counterparts, they lack allosteric regulation and are regulated solely through competitive inhibition by UTP at the active site, with catalysis proceeding via an end-to-end mechanism (Marco-Marín et al., 2005; Jensen et al., 2007).

Previous studies have elucidated the allosteric regulation and catalytic mechanism of Gram-negative bacterial UMPKs (Briozzo et al., 2005; Evrin et al., 2007; Meyer et al., 2008; Chu et al., 2012; PDB: 8YH1). The research on Gram-positive bacterial UMPKs still faces major limitations: (1) The conformational changes induced by effector binding (GTP/UTP/ATP) at the allosteric site have yet to be systematically studied. (2) Conflicting models have been proposed for UTP-mediated inhibition: one study in *Bacillus anthracis* suggests inhibition occurs at the active site (Meier et al., 2008), while another study in *Mycobacterium tuberculosis* supports binding at the allosteric site (Walter et al., 2022). (3) There is a lack of structural evidence for ATP binding at the phosphoryl donor site. (4) The role of GTP and UTP as weak phosphoryl donors remains controversial: one study reported GTP as an effective donor while UTP is not (Labesse et al., 2011), while another study found UTP effective but not GTP (Rostirolla et al., 2011). (5) Inhibitor design targeting UMPKs faces bottlenecks due to the lack of reliable structural data, with most strategies relying on computational modeling (Doig et al., 2013; Suthi et al., 2023).

To address these limitations, we determined the crystal structures of saUMPK in complex with various nucleotides, complemented by isothermal titration calorimetry (ITC) and enzymatic assays. Analysis of these results revealed several key features. In the saUMPK-UMP structure, an apo-like conformation was captured, and key residues responsible for UMP recognition were identified. The saUMPK-UTP structure further showed that UDP occupies the ATP donor site in a conformation similar to ATP binding observed in Gram-negative bacterial UMPKs, highlighting the plasticity of this site in substrate recognition. The allosteric site, enriched with basic residues, can structurally accommodate diverse nucleotides. Moreover, the type and occupancy of bound nucleotides cooperatively drive global conformational changes of the saUMPK hexamer. Site-directed mutagenesis validated the essential roles of these key residues in enzymatic activity. Collectively, these findings bridge a critical gap in understanding the structure-function relationship of saUMPK and provide a rational framework for the future development of specific inhibitors targeting saUMPK.

## 2 Materials and methods

### 2.1 Recombinant plasmid construction

The wild-type *PyrH* gene encoding wild-type saUMPK (residues 1–240, Uniprot ID: Q2FZ22) was PCR-amplified from the cDNA library of *Staphylococcus aureus* NCTC 8325 and cloned into the pET28a vector using *Nde*I and *Xho*I restriction sites. The expression construct included an N-terminal hexahistidine (His6) tag for affinity purification. Site-directed mutagenesis was performed using a PCR-based method, described in section 2.6. Primer sequences for wild-type and mutant constructs are listed in Supplementary Table 1.

### 2.2 saUMPK protein expression and purification

The recombinant expression plasmid pET28a-His6-*PyrH* encoding wild-type saUMPK was transformed into *Escherichia coli* Rosetta (DE3) strain. A single colony was inoculated into LB broth supplemented with 100 mg/L kanamycin sulfate and incubated at 37°C with shaking. The culture was scaled up stepwise until the LB volume reached 1 L. When the culture reached an OD_600_ of 0.8, protein expression was induced with 0.5 mM isopropyl β-D-thiogalactoside (IPTG) at 16°C for 20 h. Cells were harvested by centrifugation at 8,000 rpm for 6 min at 8°C, then resuspended in buffer A (500 mM NaCl, 40 mM Tris-HCl, pH 8.0) and lysed by sonication in an ice bath. After sonication, the cell lysate was centrifuged at 12,000 rpm for 30 min at 4°C and the supernatant was loaded onto a 5 mL Ni-NTA column (GE Healthcare, United States) for affinity purification. The His-tagged saUMPK was eluted from the column using buffer A containing 300 mM imidazole. Next, the eluted protein was concentrated and further purified on a 120 mL Superdex 16/200 size exclusion column (GE Healthcare, United States) equilibrated with buffer B (200 mM NaCl, 40 mM Tris-HCl, pH 8.0). Finally, the purified saUMPK protein was concentrated and stored at −80°C. The mutant proteins were purified following the same protocol as the wild-type. Typical purification results of saUMPK protein are presented in Supplementary Figure 1.

### 2.3 Crystallization, data collection, and structure determination

Crystal screening for saUMPK in both the apo form and in complex with various nucleotides (2 mM) was carried out using protein samples at 9 mg/mL dissolved in buffer B. All reagents, including those for subsequent experiments, were obtained as analytical-grade powders from Sangon Biotech (Shanghai) and dissolved in the appropriate buffer solutions to prepare working concentrations. Initial crystallization trials were performed at 16°C using the sitting-drop vapor diffusion method, by mixing 1 μL of the protein–nucleotide solution with 1 μL of reservoir solution from commercial crystal screens, including PEG/Ion, Crystal Screen, SaltRx, and Index (Hampton Research), as well as ProPlex (Molecular Dimensions). For most crystal hits, further optimization was attempted using the Additive Screen (Hampton Research).

Suitable crystals for X-ray diffraction experiments were obtained after 1 week. Diffraction data for the saUMPK-UMP and saUMPK-GTP crystals, grown in SaltRx1-29 (2.0 M sodium formate, 0.1 M Tris, pH 8.5) and SaltRx2-33 (0.7 M ammonium tartrate dibasic, 0.1 M Tris, pH 8.5), respectively, were collected at the Life Science Experimental Center of the University of Science and Technology of China. Diffraction datasets for the saUMPK–UDP, saUMPK–UTP, saUMPK–ATP/UMP, and saUMPK–ATP/GTP complexes were collected at the BL10U2 beamline of the Shanghai Synchrotron Radiation Facility (SSRF) under the following crystallization conditions: PEG/Ion 2 Screen-22 (0.2 M ammonium citrate tribasic, pH 7.0, 20% w/v polyethylene glycol 3350); SaltRx1-17 (1.0 M ammonium citrate tribasic, pH 7.0, 0.1 M BIS-TRIS propane, pH 7.0) plus 0.01 M ATP; Index-88 (0.2 M ammonium citrate tribasic, pH 7.0, 20% w/v polyethylene glycol 3350) plus 2 mM MgCl_2_; and SaltRx2-33 (0.7 M ammonium tartrate dibasic, 0.1 M Tris, pH 8.5) plus 0.01 M ATP. Attempts to obtain diffraction-quality apo-form saUMPK crystals were unsuccessful due to poor resolution (> 9 Å) despite optimization.

We processed and refined the crystal diffraction data using XDS (Kabsch, 2010), autoPX (Wang et al., 2022), CCP4 (Winn et al., 2011), Phenix (Adams et al., 2010), and Coot (Emsley et al., 2010) software. All the structures were solved by molecular replacement using *Streptococcus pyogenes* UMPK (PDB ID: 1Z9D) as the starting model. For saUMPK-UMP and saUMPK-GTP complexes, data were processed by the four-circle diffractometer software CrysAlisPro (Gul et al., 2023). The stereochemistry of the structural models was validated using the PROCHECK program (Laskowski et al., 1993). Final structural representations were visualized with PyMOL (DeLano and Lam, 2005) and LigPlot + (Laskowski and Swindells, 2011). The X-ray diffraction data refinement statistics are summarized in Supplementary Table 2.

### 2.4 Isothermal titration calorimetry binding assays

Isothermal titration calorimetry (ITC) experiments were performed using a MicroCal PEAQ-ITC instrument at 20°C at the Life Science Experimental Center of the University of Science and Technology of China. saUMPK protein (400μM in buffer B) was titrated with nucleotides (1mM each) dissolved in the same buffer, including ATP, UDP, GTP, UTP, and nucleoside monophosphates (NMPs: UMP, AMP, CMP, GMP). For competitive binding assays, GTP was supplemented at an additional concentration of 100 μM.

### 2.5 Enzymatic activity assay

saUMPK activity detection relies on luciferase fluorescence based on ATP, where luminescence intensity is proportional to ATP concentration within a linear relationship. The assay quantifies ATP consumed by the kinase reaction by measuring residual ATP. We used the Kinase-Lumi™ Max Luminescent Kinase Assay Kit (Beyotime), detecting ATP from 0 to 200 μM. The standard ATP (0.5 mM) provided in the kit was gradient-diluted in buffer (200 mM NaCl, 40 mM HEPES, pH 7.0) to concentrations of 0, 1, 2, 4, 8, 20, 40, 80, 120, 160, and 200 μM. Subsequently, an equal volume (50 μL) of the detection solution was added and mixed. Chemiluminescence was measured via the endpoint method using the SpectraMax iD5 at the Life Science Experimental Center of the University of Science and Technology of China, yielding relative luminescence units (RLU). The ATP standard curve was constructed using mean values from triplicate measurements after subtraction of the blank control at 0 μM ATP, with detailed data presented in Supplementary Figure 2. All subsequent experiments were performed in three independent biological replicates to ensure reproducibility.

Enzyme activity assays were performed in a 30°C water bath for 40min using 26ng/μL saUMPK (wild-type and mutants). The pH values for determining optimal activity were chosen based on the isoelectric point of saUMPK (pI = 5.99). For non-kinetic assays, reaction mixtures contained 400μM MgCl_2_ (or other metal ions as indicated), 100μM GTP as activator, and 200μM substrates (ATP and NMPs). Ricinine (10 mM) partially precipitated during the reaction due to poor aqueous solubility. As a double-substrate allosteric enzyme, saUMPK exhibits Michaelis–Menten kinetics with respect to the acceptor UMP and Hill-type kinetics with respect to the donor ATP. To assess the effects of substrates and effectors, two concentration gradients were tested: 1) ATP fixed at 200μM with UMP varying from 0 to 500μM (0, 20, 40, 60, 80, 100, 120, 140, 160, 180, 200, 250, 300, 400, 500μM) in the presence of 100μM GTP; 2) UMP was fixed at 200μM, while ATP was varied from 0 to 300μM (0, 20, 40, 60, 80, 100, 120, 140, 160, 180, 200, 250, 300μM) in the presence of different effectors. Three experimental conditions were tested: effector-free control, 100μM GTP (activator), and 50μM UTP (inhibitor).

### 2.6 Site-directed mutagenesis

Key functional residues of saUMPK were selected for site-directed mutagenesis based on structural analysis of its nucleotide-binding sites and protein–protein interaction interfaces. Mutagenesis was carried out using a PCR-based method with the wild-type plasmid as the template. Primer sequences for both wild-type and mutant constructs are listed in Supplementary Table 1. After PCR amplification, the methylated template DNA was digested with *Dpn*I at 37°C for 1h. The digested PCR products were then used to transform *E. coli* DH5α cells, and all mutations were confirmed by DNA sequencing.

## 3 Results

### 3.1 Protein purification and enzyme activity of saUMPK

**FIGURE 1:**
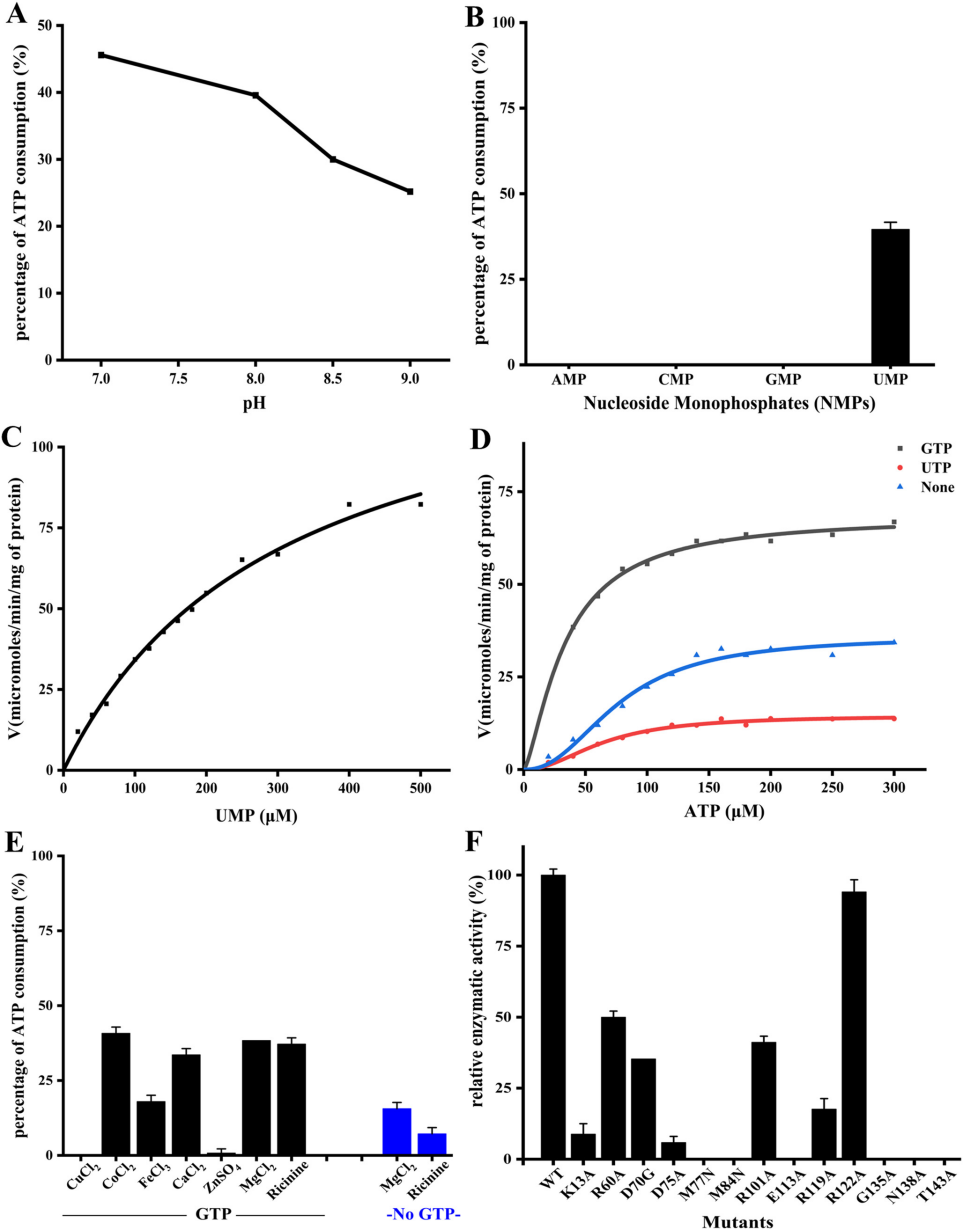
Enzymatic activity of saUMPK. **(A)** Optimal pH determination. **(B)** Validation of phosphate acceptor substrate specificity. For both **(A)** and **(B)**, the ATP consumption rate was measured. **(C)** Enzymatic reaction rate at varying UMP concentrations in the presence of GTP. The saturation plot [*V* vs. (UMP)] exhibits a characteristic Michaelis–Menten hyperbolic curve. **(D)** Enzymatic reaction rates at varying ATP concentrations, without effector (None, blue), with GTP activator (GTP, black), or with UTP inhibitor (UTP, red), following the Hill equation. **(E)** Effects of metal ions (Mg^2+^ as natural cofactor) and ricinine on enzymatic activity, assessed with or without GTP. **(F)** Relative enzymatic activities of key residue mutants compared to wild-type (WT), quantified by ATP consumption.

saUMPK is an allosteric enzyme whose activity is regulated by ligand binding at both allosteric and catalytic sites. For structural and functional analyses, saUMPK was heterologously expressed in *E. coli* Rosetta (DE3) and purified to homogeneity by sequential affinity and size-exclusion chromatography, as described in the Materials and Methods section. Enzymatic assays determined that saUMPK exhibits optimal activity at pH 7.0 (Figure 1A) and exclusive substrate specificity for UMP under the tested conditions (Figure 1B). For the two substrates of saUMPK, UMP follows Michaelis–Menten kinetics, whereas ATP displays positive cooperativity as described by the Hill equation (Figures 1C,D), consistent with previous reports (Evrin et al., 2007). GTP enhances catalysis by decreasing the *K*_0.5_ and increasing the *V*_max_ of ATP, confirming its role as a potent allosteric activator, whereas UTP acts as an allosteric inhibitor, reducing the *V*_max_ by approximately 59% (Table 1). The enzymatic activity was strongly dependent on divalent and trivalent metal ions: Co^2+^ supports enzyme activity similarly to Mg^2+^, whereas Fe^3+^, and Ca^2+^ markedly inhibit activity, and Zn^2+^ and Cu^2+^ nearly abolish it (Figure 1E). These results establish saUMPK as an allosteric enzyme whose catalytic activity is modulated in response to different nucleotides, consistent with observations reported for UMPKs from other species (Evrin et al., 2007).

**TABLE 1:** Kinetic parameters of saUMPK under different experimental conditions.

Condition	*K*_0.5_ or *K*_*m*_ (μM)	*V*_max_ (μM/min/mg)
Variable [ATP], Fixed [UMP]	77.17 ± 5.48	35.83 ± 1.86
+ Activator GTP	33.68 ± 1.55	68.40 ± 1.71
+ Inhibitor UTP	64.23 ± 4.02	14.56 ± 0.59
Variable (UMP), Fixed (ATP), + GTP	303.32 ± 27.00	137.27 ± 6.77

Values are presented as mean ± SD.

### 3.2 Monomeric structure of saUMPK

**FIGURE 2:**
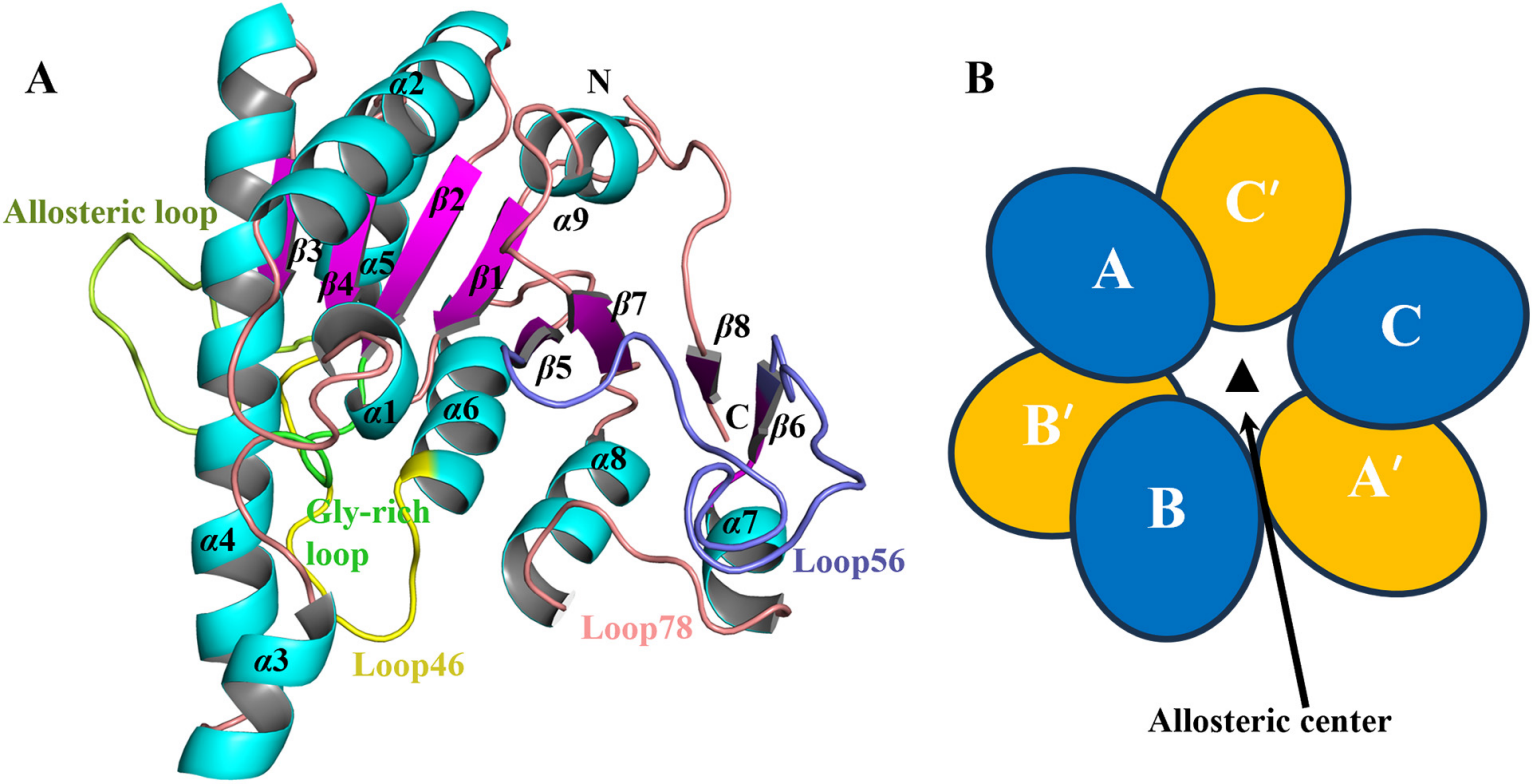
Cartoon representation of the saUMPK monomer and schematic of the hexameric assembly. **(A)** The secondary structure of the saUMPK monomer. Colors represent different secondary structure elements: α-helix, blue; β-sheet, rose. “N” denotes the N-terminus, and “C” indicates the C-terminus. The allosteric loop (residues 104–117, lemon), together with α5 and β3, forms the allosteric site for allosteric effector binding. Loop46 (residues 133–143, yellow), positioned between β4 and α6, is a component of the UMP-binding site and essential for UMP-protein interactions. Loop56 (residues 163–185, slate), connecting β5 and β6, is the longest loop of the monomeric structure, and participates in purine (pyrimidine) ring binding. A loop opposing Loop56 is called Loop78 (residues 194–201). A glycine-rich loop (green) is conserved in prokaryotic UMPKs and corresponds to the sequence motif (*54* -GGGN- *57*) in saUMPK. **(B)** Quaternary structure model of saUMPK. Viewed along the threefold crystallographic axis (represented by the triangle), the hexamer is organized with an upper set of three monomers (A, B, C) and a bottom set of three monomers (A′, B′, C′). These subunits form three dimers (AC′, BB′, CA′).

The monomeric structure of saUMPK adopts an open α/β-fold conformation, comprising nine α-helices, eight β-strands, and several connecting loops (Figure 2A). Each monomer contains a catalytic center (comprising the UMP-binding site and the ATP donor site) and a distinct allosteric site. The allosteric site is formed by α5 (together with α5′ from the adjacent monomer), β3, and the allosteric loop (residues 104–117), and is responsible for the effector binding. The UMP-binding site includes the loop connecting β1 and α1, the glycine-rich loop connecting β2 and α3, Loop46 (residues 133–143), as well as α3 and part of the α4. The ATP donor site is mainly formed by the highly flexible Loop56 (residues 163–185), which exhibits significant conformational plasticity across different structures, along with contributions from Loop78 and a short segment at the C-terminal end of β1. The spatial separation of the allosteric site and catalytic center provides the structural basis for regulation of enzyme activity through allosteric ligand binding. Multiple sequence alignment across diverse species reveals that residues of the catalytic center are highly conserved in both Gram-positive and Gram-negative bacteria, whereas the allosteric site residues display substantial variability (Supplementary Figure 4), reflecting species-specific adaptive evolution of their regulatory sites. In contrast, human CMP/UMPK (PDB: 1TEV) differs markedly from bacterial UMPKs in both sequence and structure, underscoring the value of elucidating the saUMPK structure as a template for antibacterial drug development.

### 3.3 Crystal structures of saUMPK in complex with various nucleotides

To elucidate the catalytic and allosteric regulatory mechanisms of saUMPK, we determined crystal structures of saUMPK bound to various nucleotides using X-ray crystallography. All structures were solved by molecular replacement, using *Streptococcus pyogenes* UMPK (PDB: 1Z9D, 60% sequence identity) as the template. The resolved structures encompass the full target sequence (residues 1–240), except for the N-terminal His-tag and a few unresolved residues. Detailed crystallographic refinement statistics are provided in Supplementary Table 2.

**FIGURE 3:**
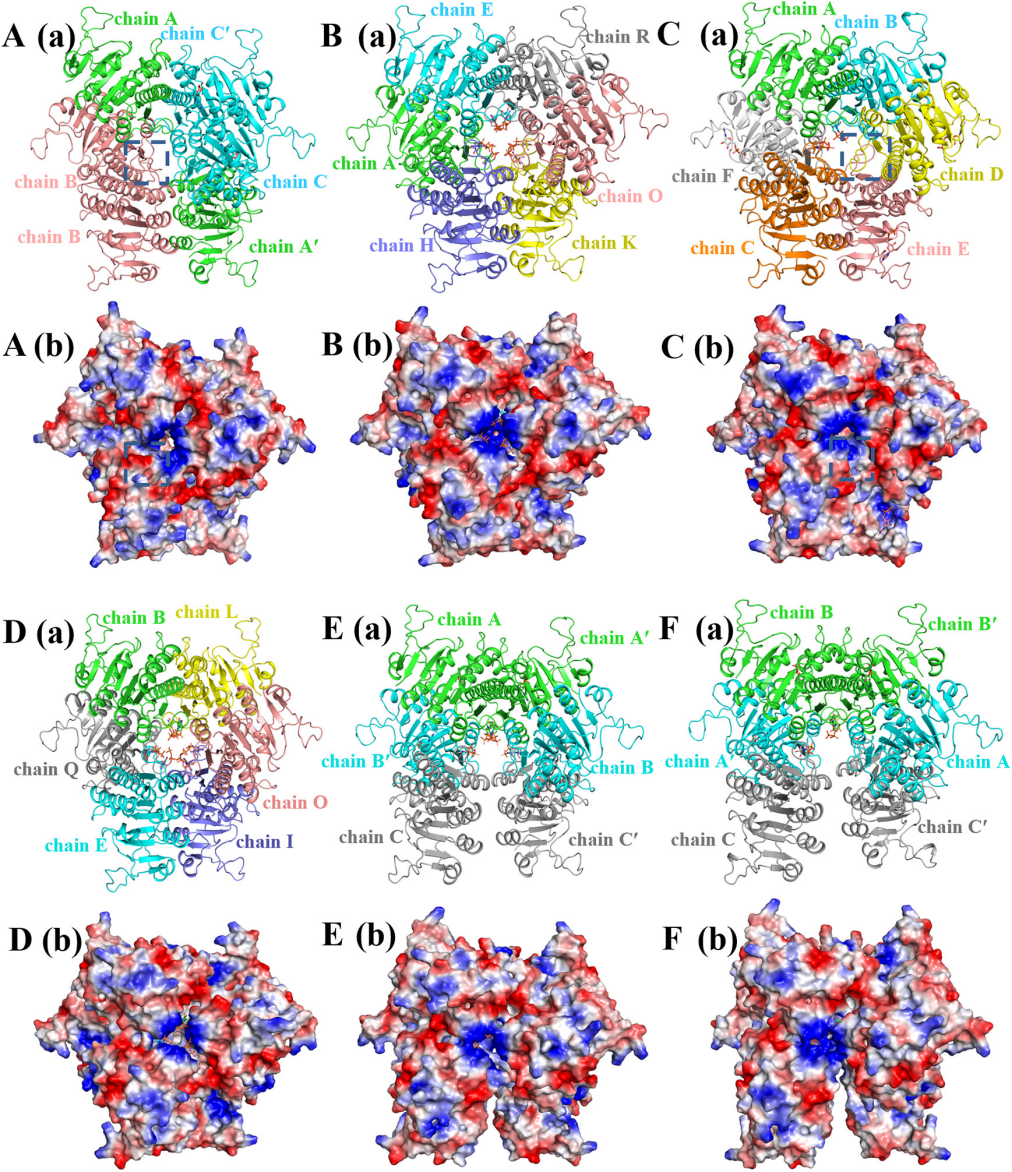
Hexameric structures and electrostatic potential distributions of saUMPK in complex with various nucleotides. **(a)** Overall hexameric structures; **(b)** Electrostatic surface potential maps (blue: positive; red: negative). Subpanels: **(A)** saUMPK-UMP, **(B)** saUMPK-GTP, **(C)** saUMPK-UTP, **(D)** saUMPK-ATP/GTP, **(E)** saUMPK-ATP/UMP, **(F)** saUMPK-UDP. The allosteric center located in the center of the structure, comprises three allosteric sites enriched with basic residues (blue patches). Allosteric sites in chain B/B′ **(A)** and chain D/E **(C)** exhibit spacious conformations with attenuated positive electrostatic potential (dashed boxes). **(D)** One hexamer from the asymmetric unit with bound ATP (green) and GTP. **(E,F)** The U-shaped hexamer has ATP (UDP) occupying two allosteric sites per allosteric center and UMP (UDP) bound at four UMP-binding sites within the U-base. Nucleotides are shown as sticks.

The determined complex structures can be classified into three categories: (1) single-substrate bound: UMP (Figure 3A) and UDP (Figure 3F); (2) single-allosteric-effector bound: GTP (Figure 3B) and UTP (Figure 3C); and (3) mixed-nucleotide bound: ATP/GTP (Figure 3D) and ATP/UMP (Figure 3E). Important structural findings include: one monomer adopts an apo-like conformation in the saUMPK–UMP complex; in the saUMPK–UTP complex, UDP (a hydrolysis product of UTP) occupies the ATP donor site, revealing the ATP donor site interactions; and in the saUMPK–UDP complex, UDP binds both the allosteric site and the UMP-binding site, resulting in a U-shaped hexameric conformation, which is also observed in the saUMPK–ATP/UMP complex structure. Detailed descriptions are presented in the following sections.

The number of monomers in the asymmetric unit varies among different complexes, likely reflecting differences in crystal packing (Supplementary Table 3). In the saUMPK–UTP structure, the asymmetric unit contains a full hexamer, whereas the saUMPK–GTP and saUMPK–ATP/GTP complex structures contain three hexamers per asymmetric unit. In contrast, the remaining saUMPK complex structures contain only a trimer in the asymmetric unit, with the biological hexamer formed through a crystallographic two-fold symmetry operation. In all structures, the hexameric assembly is either present in the asymmetric unit or can be generated by crystallographic symmetry, and it likely represents the physiologically relevant functional unit. This observation is consistent with saUMPK existing as a homohexamer in solution (Hari Prasad et al., 2012) and with similar findings reported for UMPKs from other species (Meier et al., 2008; Chu et al., 2012; Walter et al., 2022).

The saUMPK hexamer can be described as a trimer of dimers, with each dimer (AC′, BB′, or A′C) related by 3-fold symmetry, resulting in an overall assembly approximating D3 symmetry (Figure 2B). Each dimer is formed by two monomers with local two-fold symmetry, and the dimer interface is stabilized primarily through hydrophobic packing interactions (Supplementary Figure 3). Alternatively, the hexamer can also be described as a dimer of trimers: the top layer consists of an ABC trimer and the bottom layer consists of an A′B′C′ trimer, with subunits within each layer related by three-fold symmetry. Two trimers (ABC or A′B′C′) adopt staggered stacking along the vertical axis to assemble the complete hexamer. In the hexameric structure, the allosteric sites are connected and located at the central region of the hexamer (the allosteric center), where they are enriched with basic residues that facilitate binding of small nucleotides (Figure 2B). Each subunit of the hexamer also contains an ATP donor site and a UMP-binding site. Notably, nucleotide binding at the allosteric sites in the center of the hexamer induces conformational changes in each subunit, modulating cooperativity between subunits and influencing both the catalytic center conformation and overall enzymatic activity.

### 3.4 Structure of apo-like conformation of saUMPK

Analysis of the effects of nucleotides requires an apo structure. Attempts to determine the apo structure of saUMPK were unsuccessful due to conformational flexibility and poor diffraction quality (> 9 Å). ITC data revealed weak UMP binding in the absence of effectors (Supplementary Figures 5, 6). Notably, an apo-like conformation was captured in the saUMPK-UMP complex after extensive efforts in crystallization and structural analysis. In this structure, each asymmetric unit contains a trimer of saUMPK (A, B, C subunits), and the hexameric complex is formed by two asymmetric units (A, B, C and A′, B′, C′) (Figure 3A). Dimer interface analysis revealed that the AC′, CA′, and BB′ dimers exhibit strong interactions, with buried areas of ∼1,400 Å^2^, whereas other interfaces are less significant, with buried areas smaller than 700 Å^2^ (calculated by PISA). The chain A/C′ dimer, with a buried interface of 1422.1 Å^2^, binds UMP, and both Loop46 and the allosteric loop adopt a bound-state conformation. In contrast, the chain B/B′ dimer (a buried interface of 1247.8 Å^2^) lacks UMP and exhibits high conformational flexibility in the two loops. Comparison of the A and B subunits reveals a Cα root-mean-square deviation (RMSD) of 2.36 Å between their respective Loop46 and allosteric loop regions. These analyses indicate that subunit B represents an apo-like structure.

The apo-form structures of *S. pyogenes* UMPK (PDB: 1Z9D) and *H. influenzae* UMPK (PDB: 2A1F) were compared and showed that their corresponding loops adopt a bound-state conformation (Supplementary Figure 7). Sulfate ions used in crystallization interact with the allosteric sites of *S. pyogenes* UMPK and may influence its conformation.

### 3.5 Structural basis of UMP recognition at the UMP-binding site

**FIGURE 4:**
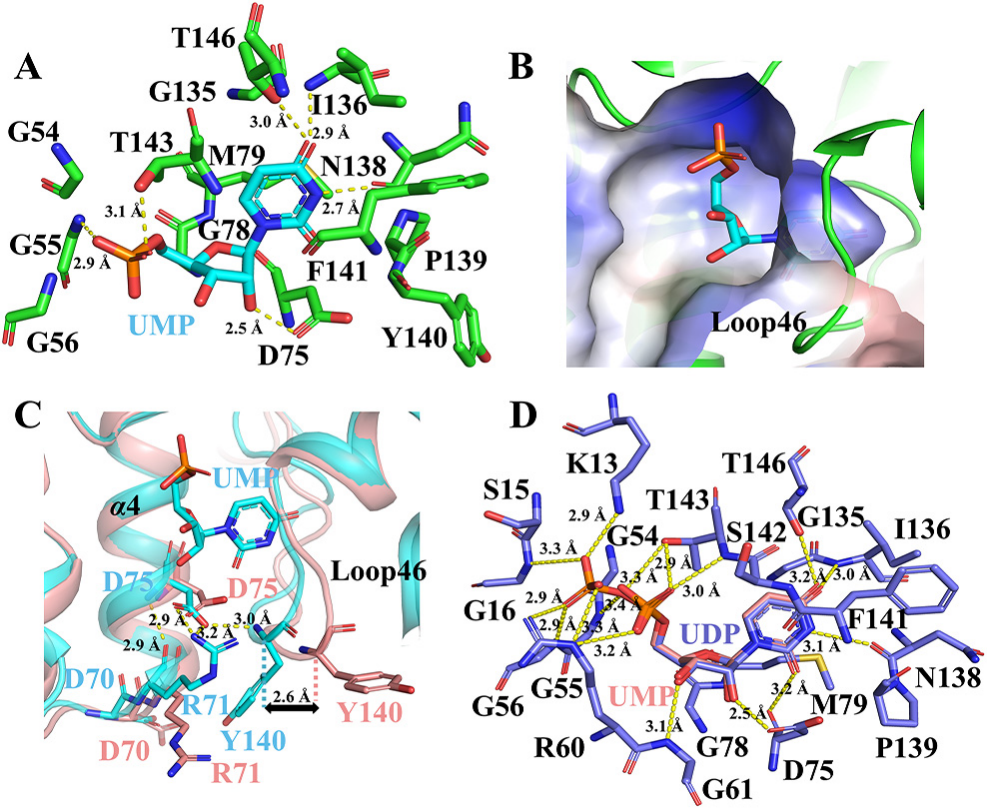
Structural basis of UMP and UDP recognition at the UMP-binding site of saUMPK. **(A)** Key interactions stabilizing UMP (blue) involve residues (green), including the conserved “GGGN” motif (G54–G56), α4 (D75, G78, M79), Loop46 (G135–T143), and α6 (T146). **(B)** Electrostatic potential map of the UMP-binding pocket; blue patches indicate positive potential. **(C)** Superposition of UMP-bound (blue) and apo-like (pink) structures highlights key conformational differences in the UMP-binding site, including the R71–D75–Y140 hydrogen-bond network (absent in the apo-like structure). An increased Cα–Cα distance of ∼2.6Å is observed between Y140 and D75 in the apo-like state compared with the bound state. **(D)** UDP (slate) forms specific interactions via its additional phosphate group and an inward shift of α3, engaging K13, G16, G56, R60, and G61. Hydrogen bonds (2.5–3.5 Å) are shown as yellow dashed lines, with their corresponding distances labeled.

The biochemical assay showed that saUMPK specifically recognizes UMP and the binding affinity for UMP increases when the allosteric site is occupied by effectors (Figure 1B; Supplementary Figures 5, 6). UMP binding in the saUMPK–UMP structure reveals the mechanism of UMP recognition at the catalytic center. The UMP-binding site is a narrow, substrate-specific pocket that engages UMP through a network of hydrogen bonds and hydrophobic interactions (Figures 4A,B). In this structure, conserved residues (I136, N138) are primarily responsible for binding to UMP’s pyrimidine ring. Importantly, the UMP-binding site features an R71–D75–Y140 hydrogen-bond network that is absent in the apo-like state (Supplementary Figure 8C). This absence results from the displacement of Loop46 from α4, which mispositions R71 and Y140 and increases the Cα–Cα distance between Y140 and D75 by approximately 2.6 Å relative to the bound state (Figure 4C). This network appears to be critical for UMP binding and catalysis, as further supported by the markedly reduced activity of the D75A mutant in biochemical assays (Figure 1F). This network is also conserved in other bacterial UMPKs (e.g., R68–D72–F138 in *B. anthracis* UMPK), and mutation of the key aspartate has confirmed its essential role in bacterial survival and growth (Lee et al., 2007). However, the regulatory role of this network in stabilizing the UMP-binding site and facilitating UMP recognition has not been recognized in previous studies.

In the saUMPK-UDP complex structure (Figure 3F), UDP binds to both the allosteric site and the UMP-binding site, which is consistent with ITC measurements showing two distinct binding constants (Supplementary Figure 9D). Residues mediating UDP binding at the UMP-binding site are conserved, especially D75, I136, N138, and T143. UDP’s β-phosphate group enables novel hydrogen bonds with K13, G16, and G56, while movement of α3 brings two additional residues, R60 and G61, to interact with UDP (Figure 4D). Comparison of saUMPK-UMP and saUMPK-UDP structures reveals that important conserved residues (D75, I136, N138, and T143) are involved in UMP recognition, and also that the allosteric loop and Loop46 adopt a bound conformation.

### 3.6 Capture of the ATP donor site in a substrate-bound state

**FIGURE 5:**
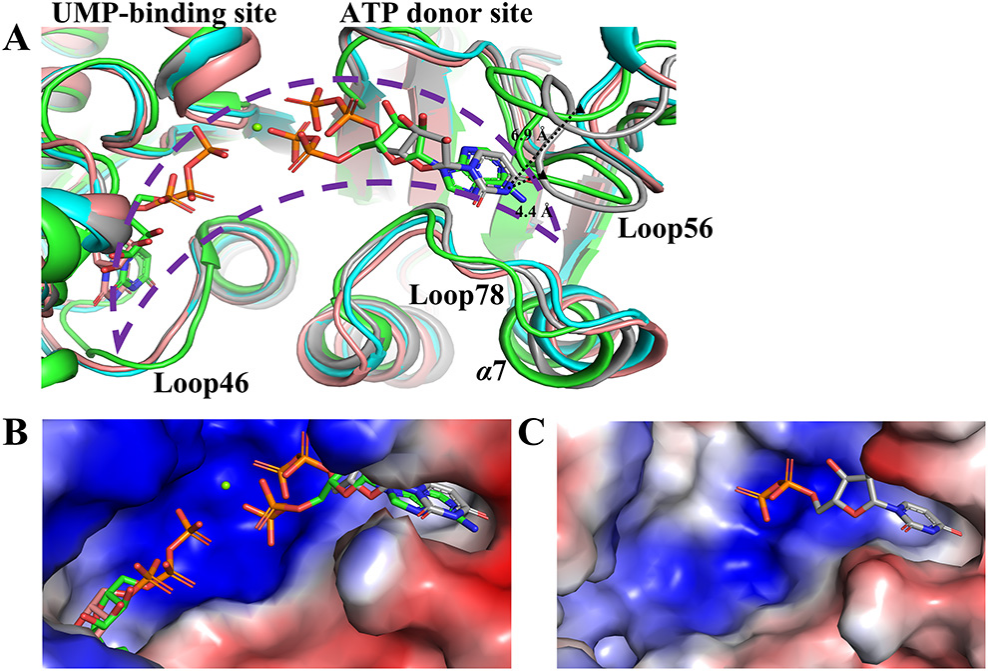
Structural and electrostatic potential comparisons of the active center in *S. aureus* versus *T. thermophilus* UMPK complexes (PDB: 8YH1). **(A)** Structural superposition of UMP-bound chain C (pink) of saUMPK-UMP, UTP-bound chain B (blue) and UDP-bound chain F (concrete grey) of saUMPK-UTP, and GTP/ADP/UDP-bound chain C (green) of *T. thermophilus* UMPK. The distance between the UDP N3 and D173 Cα decreases from 6.9 Å in the UDP-free state to 4.4 Å in the UDP-bound state where black triangle (▲) denotes the position of D173 Cα. **(B)** Electrostatic potential map (red: negative; blue: positive) of the active center in *T. thermophilus* UMPK, which is spacious and enriched in basic residues. The positions of its bound ADP and UDP overlap with those of UDP and UMP in saUMPK structures. **(C)** Electrostatic potential map of the active center in chain F of saUMPK-UTP, which is similar to that of *T. thermophilus* UMPK. Green sphere: Mg^2+^. Nucleotides are depicted as sticks (colored by chain). In the *S. aureus* active center, UDP occupies the ATP donor site with a concomitant contraction of Loop56/α7, aligning with UMP (pink) in an end-to-end catalytic mechanism (purple dashed area).

The ATP donor site in saUMPK exhibits conformational flexibility, spatial accessibility, and a cluster of positively charged residues, forming a pocket well-suited for binding polyphosphorylated nucleotides (Figure 5). In the saUMPK-UTP complex structure, two UTP molecules (one per allosteric center) were observed bound at the allosteric sites of the hexamer. Interestingly, additional electron densities appeared at the ATP donor sites, allowing the assignment of three UDP molecules, as indicated by the electron density maps. The UDP ring is embedded between Loop56 and Loop78, inducing a local conformational contraction of Loop56 and α7 (Figure 5A; Supplementary Figure 10A). This contraction reduces the distance between the UDP N3 atom and D173 Cα atom to 4.4Å, compared with 6.9Å in the UDP-free state. Moreover, structural alignment with *T. thermophilus* UMPK corroborates the positioning of the ATP donor site (Figures 5A,B). This represents the first structural evidence of the ATP donor site captured in a bound state in Gram-positive bacterial UMPKs, highlighting both the conformational plasticity of this site and the difficulty of capturing it in a ligand-bound form.

Functional assays were performed to validate the structural insights. In the single-substrate ITC assay, GTP displays two binding constants to saUMPK (Supplementary Figure 9C), suggesting occupancy at both the allosteric site and the ATP donor site, and indicating that GTP may act as an alternative phosphate donor. In contrast, UTP shows a single binding constant in the same assay (Supplementary Figure 9A), indicating predominant binding to the allosteric site. These results suggest that the ATP donor site exhibits promiscuous substrate binding and may allow various phosphate donors to participate in end-to-end catalysis with UMP (Figure 5A).

### 3.7 Recognition of different nucleotides at the allosteric site of saUMPK

**FIGURE 6:**
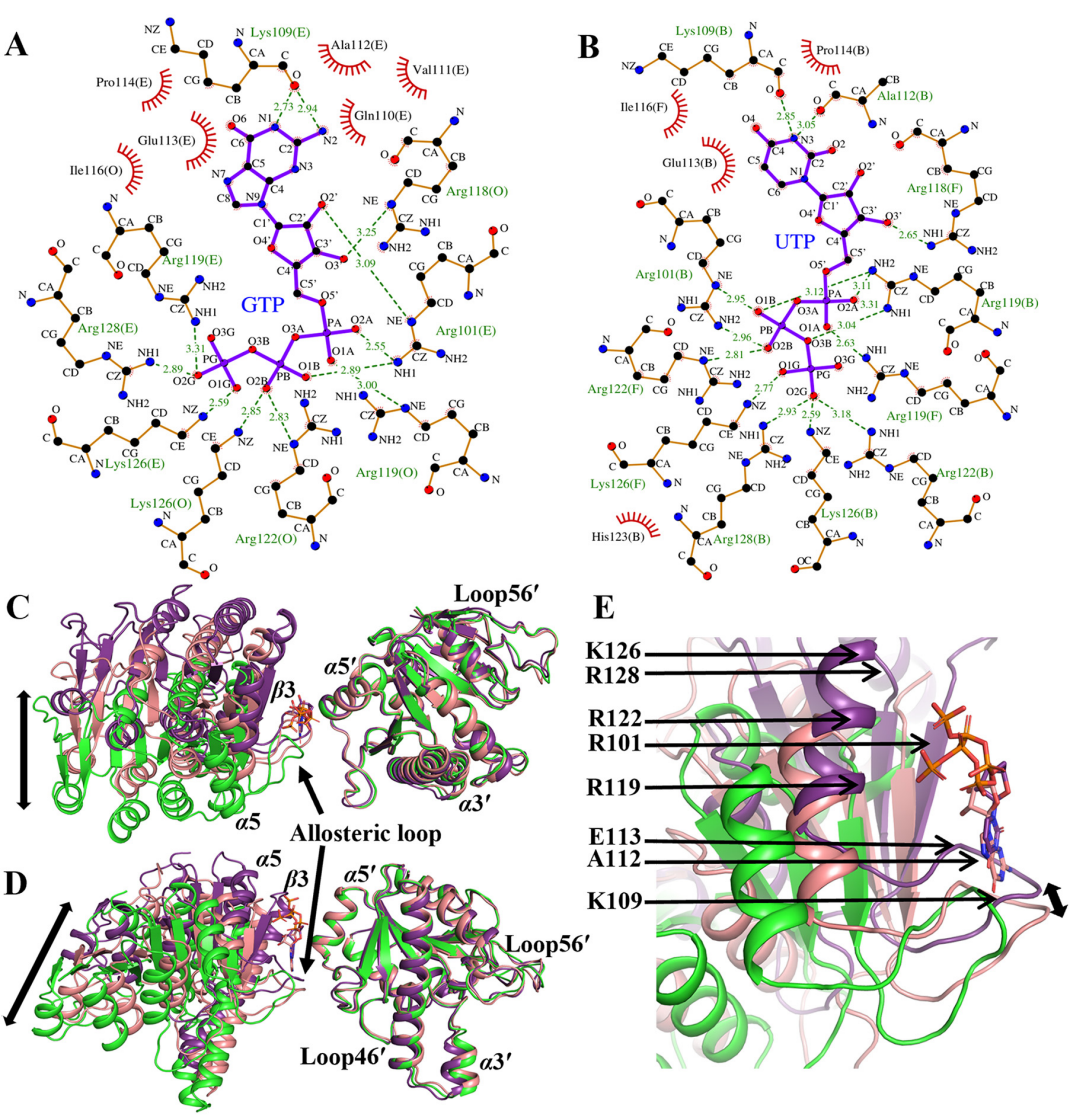
Recognition of GTP and UTP binding at the allosteric sites of saUMPK and corresponding conformational rearrangements. **(A,B)** LigPlot + diagrams depicting interactions of the saUMPK allosteric sites with GTP **(A)** or UTP **(B)**. Atom colors: red (O), black (C), and blue (N). Hydrogen bonds (2.5–3.5 Å) are depicted as green dashed lines with distances labeled. Hydrophobic interactions are marked by red arcs. **(C–E)** Conformational rearrangements induced by GTP or UTP binding in non-dimerized adjacent monomers that form the complete allosteric site (comprising α5, α5′, β3, and the allosteric loop). Structural models are colored as UTP-bound (deep purple), GTP-bound (pink), and apo-like (green). Superpositions are shown relative to the apo-like state, with the α5′-containing monomer fixed. **(C)** Top view. **(D)** Front view. **(E)** Expanded view of the allosteric loop region (**D**, left monomer) with key interacting residues labeled. Nucleotides are shown as sticks. Black double-headed arrows indicate the direction of monomer conformational movements.

The crystal structures of the saUMPK-GTP and saUMPK-UTP complexes (Figures 3B,C) reveal the structural basis of allosteric regulation. Both effectors bind to the allosteric site through a conserved hydrogen-bond network formed by key basic residues R101, K109, R118, R119, R122, K126, and R128 (Figures 6A,B). Within this network, K109 anchors the N atom of the pyrimidine or purine ring, R118 interacts with the ribose group, and the remaining residues cooperatively stabilize the phosphate groups. Furthermore, the allosteric loop participates in local hydrophobic interactions involving residues A112, E113, P114, and I116, with A112 forming a hydrogen bond to UTP or maintaining hydrophobic contacts with GTP.

GTP molecules occupy all allosteric sites with a total of six GTP molecules bound per hexamer (Figure 3B), inducing uniform contraction and stabilizing both the allosteric loop and Loop46 in their bound conformations. This maintains global three-fold symmetry with minimal variations among individual monomers in the hexamer (Cα RMSD ≤ 0.3 Å between two individual subunits), and GTP predominantly adopts a compact conformation (Figure 6E). In contrast, at the same concentration, two UTP molecules are bound per hexamer with each allosteric center accommodating a single UTP. Binding of UTP in an extended conformation induces pronounced upward shifts of the allosteric loop and α5, greater local contractions (Figures 6C–E), and loss of perfect three-fold symmetry (Figure 3C). Monomers within the dimers that interact with UTP adopt a consistent bound-state conformation in both the allosteric loop and Loop46 (Cα RMSD = 0.26–0.32 Å between two monomers), with subtle conformational adjustments observed for K109 in the UTP-contacting monomer (Supplementary Figure 10B). In contrast, the unbound monomers maintain an apo-like flexible conformation in their two loops (Supplementary Figure 10A) and display a weakened electrostatic potential at the allosteric site (Figure 3C). Nevertheless, they retain UMP-binding capability through conformational adjustments of R71, Y140 and neighboring residues (Supplementary Figures 6A–C). Superposition of GTP- and UTP-bound monomer structures with the apo-like structure reveals key differences (Figure 4C; Supplementary Figures 8A–C, 10A): an N-terminal shift of α2 toward the dimer interface; a clockwise rotation of R60 toward the UMP-binding site; the adoption of a bound conformation by the allosteric loop and Loop46; and the maintenance of an open active site with Loop56/α7 contraction and the R71–D75–Y140 hydrogen-bond network formation. These changes reflect the conformational rearrangements induced by GTP or UTP upon binding at the allosteric sites.

**FIGURE 7:**
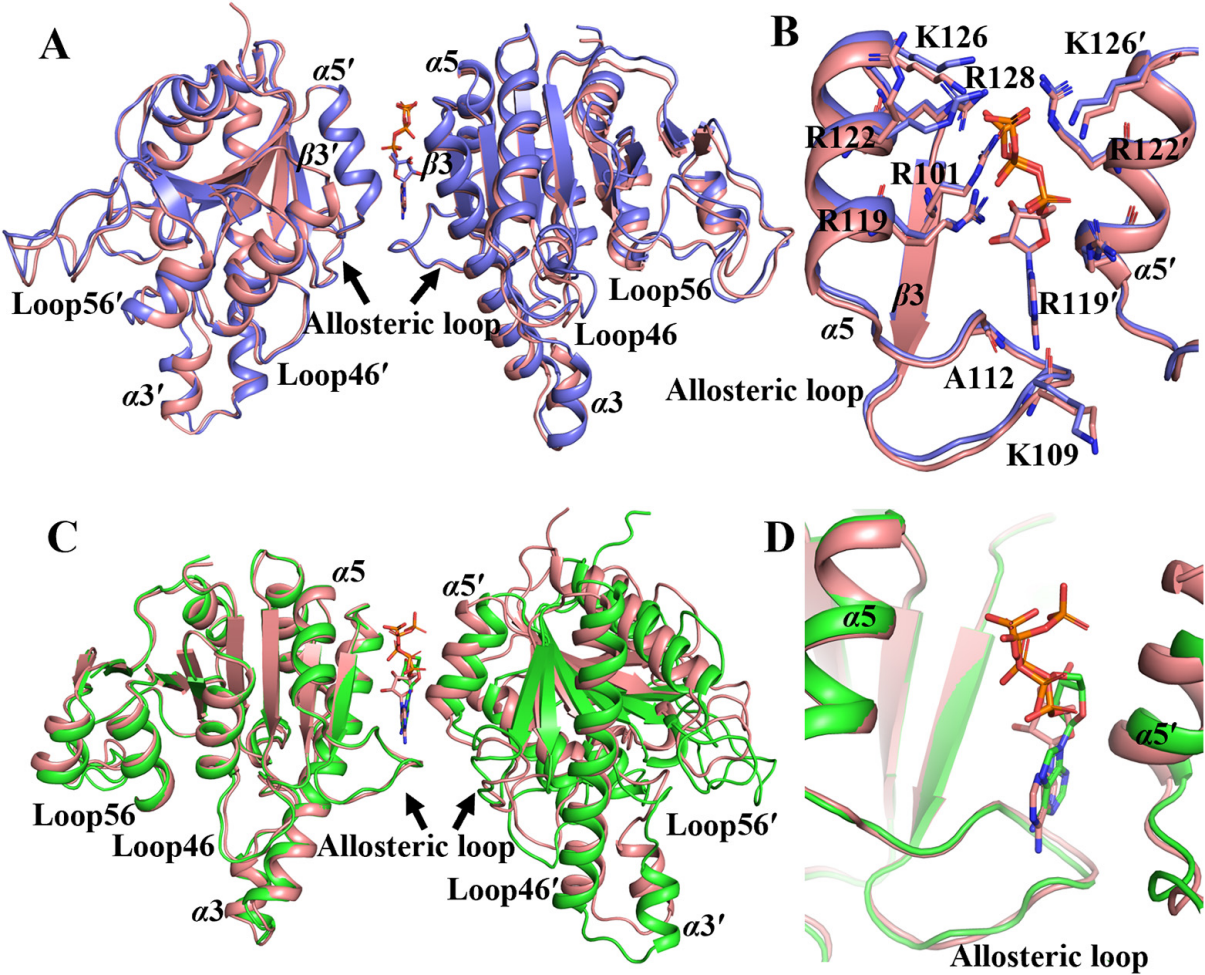
Structural comparisons of the ATP- versus UTP-bound states, and of two distinct ATP-bound states, in saUMPK. Each structure, derived from non-dimerized adjacent monomer pairs, forms a complete allosteric site. **(A,B)** Structural superposition between the saUMPK-ATP/UMP and saUMPK-UTP complexes. **(A)** Front view. **(B)** Expanded view of the complete allosteric site (rotated 180^^°^ from A), with key residues for ATP and UTP binding labeled. **(C,D)** Structural superposition of saUMPK in two distinct ATP-bound conformations: the saUMPK-ATP/UMP complex (exhibiting a U-shaped hexamer), and the saUMPK-ATP/GTP complex. **(C)** Front view. **(D)** Expanded view of the allosteric site. Structures are colored as follows: saUMPK-ATP/UMP, pink; saUMPK-ATP/GTP, green; saUMPK-UTP, slate. Nucleotides are shown as sticks (colored by chain).

Besides GTP and UTP, the donor ATP can also function as an allosteric effector. Structures of the saUMPK-ATP/GTP and saUMPK-ATP/UMP complexes were determined to investigate this effector role of ATP (Figures 3D,E). In the saUMPK-ATP/GTP complex (Figure 3D), each asymmetric unit consists of three saUMPK hexamers. GTP molecules preferentially occupy and stabilize allosteric sites via higher affinity interactions (Supplementary Figures 9B,C, 11), forming a rigid hexameric scaffold with low *B*-factors (Supplementary Table 2). ATP fills remaining sites with lower occupancy and conformational heterogeneity (Figures 7C,D; Supplementary Figure 12), suggesting ATP binding to the allosteric site is less favorable or weaker than GTP. This is consistent with ATP’s primary role as a phosphate donor. All nucleotides bind via the aforementioned conserved residues, maintaining active sites in the open conformation.

In the saUMPK–ATP/UMP complex, each asymmetric unit consists of a trimer (subunits A, B, and C), and the full hexameric assembly is generated by two such asymmetric units through a two-fold symmetry operation. Notably, this hexamer adopts a unique U-shaped closed conformation (Figure 3E), resembling the UDP-bound structure (Figure 3F) (Cα RMSD = 0.33 Å between two trimers, Supplementary Figure 13). Beyond the allosteric loop and Loop46 adopting a bound conformation, subunits A and B in the saUMPK-ATP/UMP complex undergo substantial α3 displacement, resulting in a closed active site that encloses UMP (Supplementary Figures 8D,E). At the structural level, this movement is likely facilitated by steric hindrance between P29/L67 and R60/T63 residues (Supplementary Figure 14). The remaining monomer, subunit C, retains a flexible apo-like conformation of Loop46, and its active site is solvent-exposed due to disruption of the allosteric site. Superpositions of ATP/UMP- and UTP-bound monomers, along with their respective adjacent monomers, reveal a high similarity in overall structure except for the displacement of α3 (Cα RMSD = 0.44 and 0.58 Å), and a conserved nucleotide binding mode at the allosteric site (Figures 7A,B). Collectively, these structural comparisons suggest that saUMPK hexamer bound to ATP or UDP adopts a U-shaped closed conformation that resembles the catalytically inhibited state previously observed in the *M. tuberculosis* UMPK-UTP/UDP structure (Walter et al., 2022).

In summary, nucleotide binding induces contraction of allosteric sites and an overall upward shift of monomers, facilitating adaptation to the spatial effects induced by different effectors. They drive conformational differentiation of the hexamer depending on the type and occupancy of bound nucleotides. Notably, partial occupancy of allosteric sites by ATP or UDP induces a U-shaped closed conformation, revealing that UTP is not the sole trigger of this structural transition in Gram-positive bacteria.

### 3.8 Functional validation of key saUMPK residues via site-directed mutagenesis

To assess the roles of the interface and substrate-binding sites in catalysis, key residues were subjected to site-directed mutagenesis, and the enzymatic activities of the purified mutant proteins were subsequently evaluated (Figure 1F). The dimer interface is primarily stabilized by the longest helix α4 (residues 70–97; Supplementary Figure 3), which features a hydrophobic core (M77, M84) and terminal hydrogen-bonding interactions between D70–D91 and L67–Q95, collectively contributing to interface stability. Mutations of the hydrophobic core residues (M77N and M84N) disrupt the stability of the dimer interface, causing complete loss of enzymatic activity. D70 also participates in the non-canonical interface and lies within a conformationally stable region (Supplementary Figure 15), whose mutation to glycine (D70G) results in a partial reduction of enzymatic activity.

The allosteric site contains numerous basic residues that coordinate nucleotide binding, including R101, R119, R122, K126, and R128. The R122A mutation exhibits only a minimal effect on enzymatic activity. R101, which mediates phosphate group interactions across all binding sites, and R119, which helps position the phosphate groups (Figure 7B; Supplementary Figure 13B), are relatively critical; mutations at either residue impair enzymatic activity. The unique acidic residue E113, located within a basic cluster and important for local conformational stability, is essential for enzymatic function; the E113A mutation completely abolishes activity. The integrity of the UMP-binding pocket is essential for catalysis. Residue N138, located in Loop46, contributes to uracil recognition and engages in non-canonical interface interactions with I136, functionally analogous to the T138/N140 residues reported in *E. coli* UMPK (Briozzo et al., 2005). G135 and T143 residues directly interact with UMP. Mutations N138A, G135A (introducing steric hindrance), and T143A completely abolish enzymatic activity. In addition, mutation of the highly conserved residue D75, which coordinates UMP and serves as a central hydrogen-bond hub (Figure 4), results in severely impaired enzymatic activity. Mutation of R60, which also engages the phosphate group, retains partial activity, indicating a supportive but non-essential role in catalysis. Notably, in *Vibrio vulnificus* UMPK, the R62H/D77N double mutant (corresponding to R60 and D75 in saUMPK) impairs bacterial viability (Lee et al., 2007), further underscoring the evolutionarily conserved role of the UMP-binding site in enzyme catalysis and bacterial survival. K13, located in the basic patch of the active center, is critical for ATP binding, phosphate transfer, and maintaining charge distribution (Figure 4D; Supplementary Figure 16; Briozzo et al., 2005; Jensen et al., 2007; Rostirolla et al., 2011). The K13A mutation markedly reduces catalytic activity.

In summary, mutagenesis confirms that both interface stability and the integrity of the conserved catalytic center are essential for enzymatic activity. In contrast, allosteric sites largely maintain regulatory plasticity via a network of redundant residues.

## 4 Discussion

As a key allosteric enzyme in bacterial pyrimidine biosynthesis, saUMPK regulates nucleotide metabolic flux, influencing pathogen adaptability and survival, and thus represents a promising antibacterial drug target. Our structural and biochemical analyses reveal: (1) a unique apo-like conformation; (2) a conserved UMP recognition mechanism; (3) substrate-binding plasticity at the ATP donor site; and (4) a conserved network of basic residues mediating nucleotide binding at the allosteric sites to regulate hexamer conformation. Combined with functional data, these findings elucidate the structure-function relationship of saUMPK, demonstrate how nucleotide binding drives conformational differentiation, and provide a structural basis for designing broad-spectrum antibacterial drugs targeting UMPK.

Apo-form UMPK structures (PDB: 1Z9D, 2A1F) have been reported previously, in which the allosteric loop and Loop46 adopt a bound conformation. In contrast, the apo-like conformation observed in saUMPK–UMP reveals a concerted relaxation of these two loops, representing a previously unrecognized feature of allosteric regulation in Gram-positive bacteria. This conformation may result from the absence of ionic-binding interference and likely represents the initial, unliganded state of saUMPK. These observations help explain the challenges associated with resolving apo saUMPK structures using X-ray crystallography, as the relaxed conformation captured here resembles an apo state relative to previously reported bacterial UMPK structures.

Allosteric regulation of UMPK by nucleotides (UTP/GTP/ATP) occurs through differential occupancy of the allosteric sites. Partial occupancy by the inhibitor UTP induces a U-shaped hexamer associated with catalytic inhibition (Walter et al., 2022), whereas full occupancy by GTP or ATP results in a globally symmetric, open hexameric conformation (Meier et al., 2008; Labesse et al., 2011). These observations support a model in which both the type and number of bound effectors dictate the overall hexameric conformation. Our results corroborate and extend this model. In the saUMPK-GTP or saUMPK-ATP/GTP structure, the allosteric sites are fully occupied, and the hexamer conformation resembles that reported in previous studies (Meier et al., 2008; Labesse et al., 2011). ATP and GTP bind simultaneously to allosteric sites, exhibiting a significant difference in binding stoichiometry and a unique conformation of ATP. This may be attributed to the higher binding affinity of GTP and greater conformational constraint, leading to a deviation in the ATP-bound conformation. Surprisingly, the hexameric structure of the saUMPK-ATP/UMP complex is U-shaped, unlike that in *B. anthracis* (Meier et al., 2008); this difference may arise from the less favorable binding of ATP than that of GTP at allosteric sites or from species-specific differences, excluding any effect of UMP binding (Walter et al., 2022). This observation is consistent with the hexamer structures of the saUMPK-UTP complex (this work) and the mtUMPK-UTP/UDP complex (Walter et al., 2022). Furthermore, the allosteric site exhibits binding plasticity, accommodating UDP with higher affinity than UTP. Extending previous studies, we report for the first time that the saUMPK–UDP complex also exhibits partial occupancy of allosteric sites, with the hexamer adopting a U-shaped closed conformation. It suggests that the U-shaped conformation may optimize the steric compatibility of this site to create a high-affinity environment for nucleotides with fewer phosphate groups. This provides a new perspective on the conformational selectivity and ligand adaptability of allosteric sites.

Regardless of the conformational state of the hexamer, the catalytic center of saUMPK undergoes similar conformational fine-tuning upon different effector binding and operates via an end-to-end catalytic mechanism. The UMP-binding site is highly UMP-specific and conserved, consistent with the previous report (Rostirolla et al., 2011). In contrast, the ATP donor site has been controversial, with no structural evidence for nucleotide binding in Gram-positive bacterial UMPKs. Indeed, differences in experimental methodologies, lower enzymatic activity measurements, and the lack of comprehensive structural and biochemical evidence may have contributed to the ongoing debate regarding weak phosphate donors such as GTP or UTP (Labesse et al., 2011; Rostirolla et al., 2011) and uncertainties in the UTP-mediated inhibition model (Meier et al., 2008; Walter et al., 2022). This work provides the first structural evidence of the ATP donor site captured in a nucleotide-bound state in Gram-positive bacterial UMPKs, highlighting its remarkable conformational plasticity in accommodating different polyphosphorylated nucleotides, such as UDP. Notably, this non-canonical UDP binding mode has not been observed in the previously resolved bacterial UMPK structures, including both Gram-negative (Briozzo et al., 2005; Chu et al., 2012) and Gram-positive bacteria (Labesse et al., 2011; Walter et al., 2022), where UDP binds exclusively to the UMP-binding site. The difference may be due to species-specific variations or difficulty in capturing the ATP donor site in a ligand-bound form due to its dynamic nature.

*Staphylococcus aureus* is a highly pathogenic bacterium notable for its antibiotic resistance and biofilm formation (Gu et al., 2023; Shi, 2025). There is an urgent need for new antimicrobial agents targeting this pathogen. However, the development of saUMPK inhibitors remains limited. Only a few compounds, including ricinine (Swarupa et al., 2017), MMOXC (a ricinine derivative; Swarupa et al., 2017; Suthi et al., 2023), ATP analogs (Doig et al., 2013), and PYRH-1 (a non-substrate analog; Yoshida et al., 2012), have been reported, and none provided atomic-level structural information. Consequently, rational inhibitor design must integrate atomic-level structures, conformational dynamics, and substrate specificity, particularly when elucidating inhibition mechanisms and optimizing drug candidates, rather than relying solely on molecular docking and/or *in vitro* assays. Based on the structural insights and the elucidated catalytic and allosteric regulatory mechanisms of saUMPK, several strategies for designing potent inhibitors can be considered: (1) Allosteric site inhibitors. This category of compounds includes conformation-regulating inhibitors, such as UTP/UDP analogs, which drive the hexamer into a U-shaped closed conformation, and allosteric blockers (e.g., ricinine, which affects GTP binding as shown in enzymatic assays, Figure 1E) that competitively occupy the allosteric site to prevent other nucleotides from initiating conformation-based regulation. (2) UMP-binding site inhibitors. This group comprises competitive UMP analogs that target this site. Besides, targeting the dimeric interface may represent another valuable avenue to compromise UMPK function and bacterial viability. Other strategies to design GTP (act as an activator) or ATP analogs (complex regulation mechanism) are less desirable.

Despite the rational design strategies outlined above, several experimental limitations should be acknowledged. The small size of ricinine results in weak binding, preventing structural determination and necessitating further medicinal chemistry efforts to increase its molecular size and enhance its drug-like properties. In addition, the methodology used in enzymatic activity assays limited the exploration of weak phosphate donors. Nevertheless, the extensive structural and biochemical data presented here provide a deeper understanding of saUMPK’s regulatory mechanism and lay a solid foundation for the development of antibacterial drugs targeting *S. aureus* and related Gram-positive pathogens in the future.

## Data Availability

The structural data presented in the study can be found in the Protein Data Bank repository (https://www.rcsb.org), accession numbers 9UVK, 9UVN, 9UVO, 9UVP, 9UY1, and 9UYX.
