# Supplementary material for: Structural basis for the allosteric regulation and catalytic mechanism of Staphylococcus aureus UMP kinase
Source: Front Microbiol. 2026 Jan 14;16:1733028. doi: 10.3389/fmicb.2025.1733028 (PMC12847342; doi:10.3389/fmicb.2025.1733028)
Supplement: Supplementary file 1 [file Data_Sheet_1.pdf]

Supplementary Material

1 Supplementary Figures and Tables

1.1 Supplementary Figures

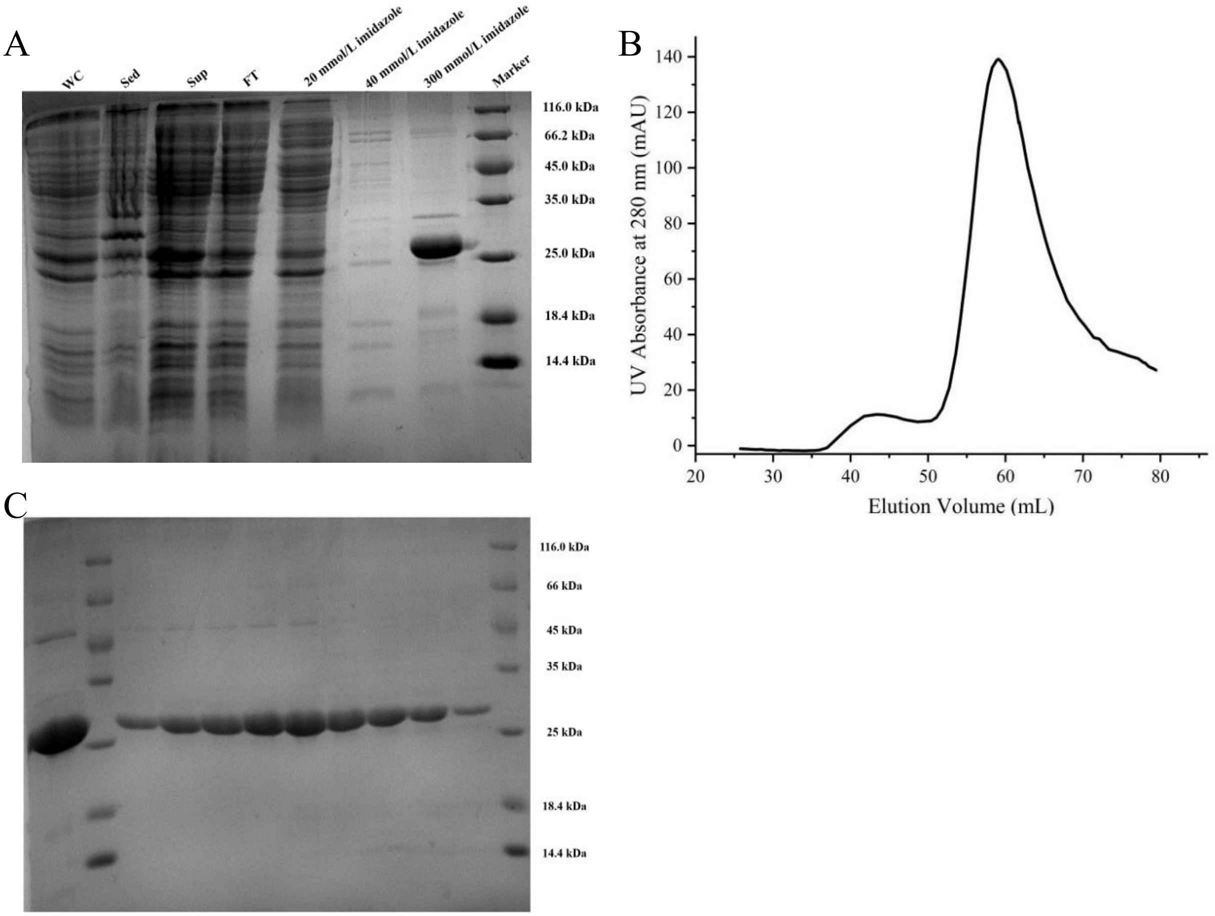

Supplementary Figure 1. Purification and chromatography of saUMPK. (A) SDS-PAGE analysis of saUMPK-enriched cell supernatant purified using a Ni^{2+} -affinity column. The rightmost lane represents the protein marker with molecular weights (kDa). The thickest band (left of the 25 kDa marker) corresponds to the saUMPK protein. (B) Chromatogram of saUMPK using a Superdex 16/200 size-exclusion column. A single dominant peak was observed during the elution process. Proteins from this peak were utilized for crystallization screening and biochemical experiments. (C) SDS-PAGE gel image of the main peak fractions from size-exclusion chromatography. The leftmost lane shows the concentrated saUMPK sample prior to gel chromatography; the second and rightmost lanes are markers.

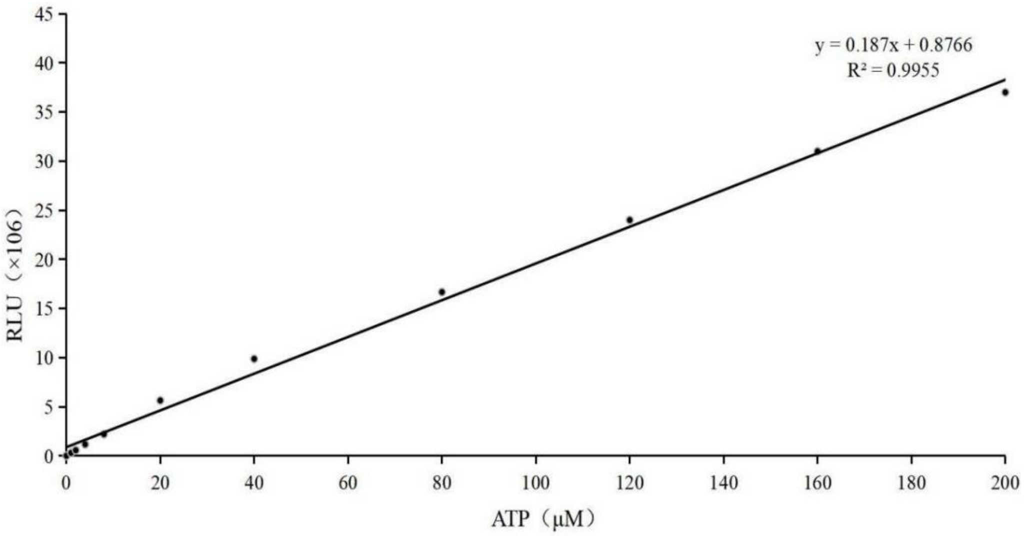

Supplementary Figure 2. The standard curve of ATP. The x-axis represents ATP concentration; the y-axis denotes relative luminescence units (RLU).

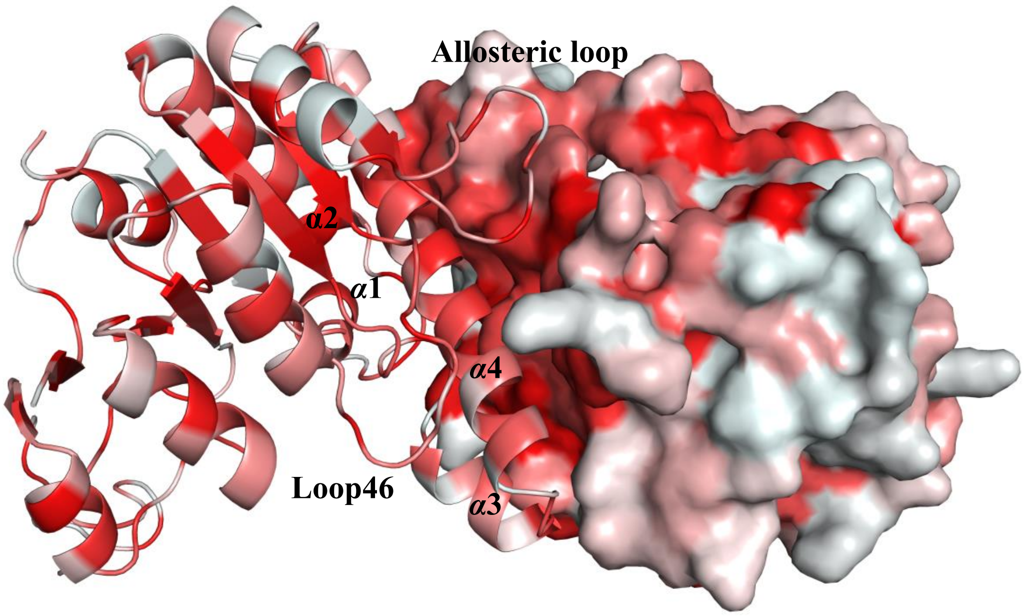

Supplementary Figure 3. Structural basis of hydrophobic interactions stabilizing the homodimer interface in saUMPK. Homodimer structure is depicted as gray ribbons with molecular surface overlaid on the right monomer. Red patches indicate hydrophobic residues. The dimerization interface, formed by the loop between *α*1 and *α*2, the *α*3 N-terminal loop–*α*4, and the allosteric loop, is primarily stabilized by hydrophobic interactions.

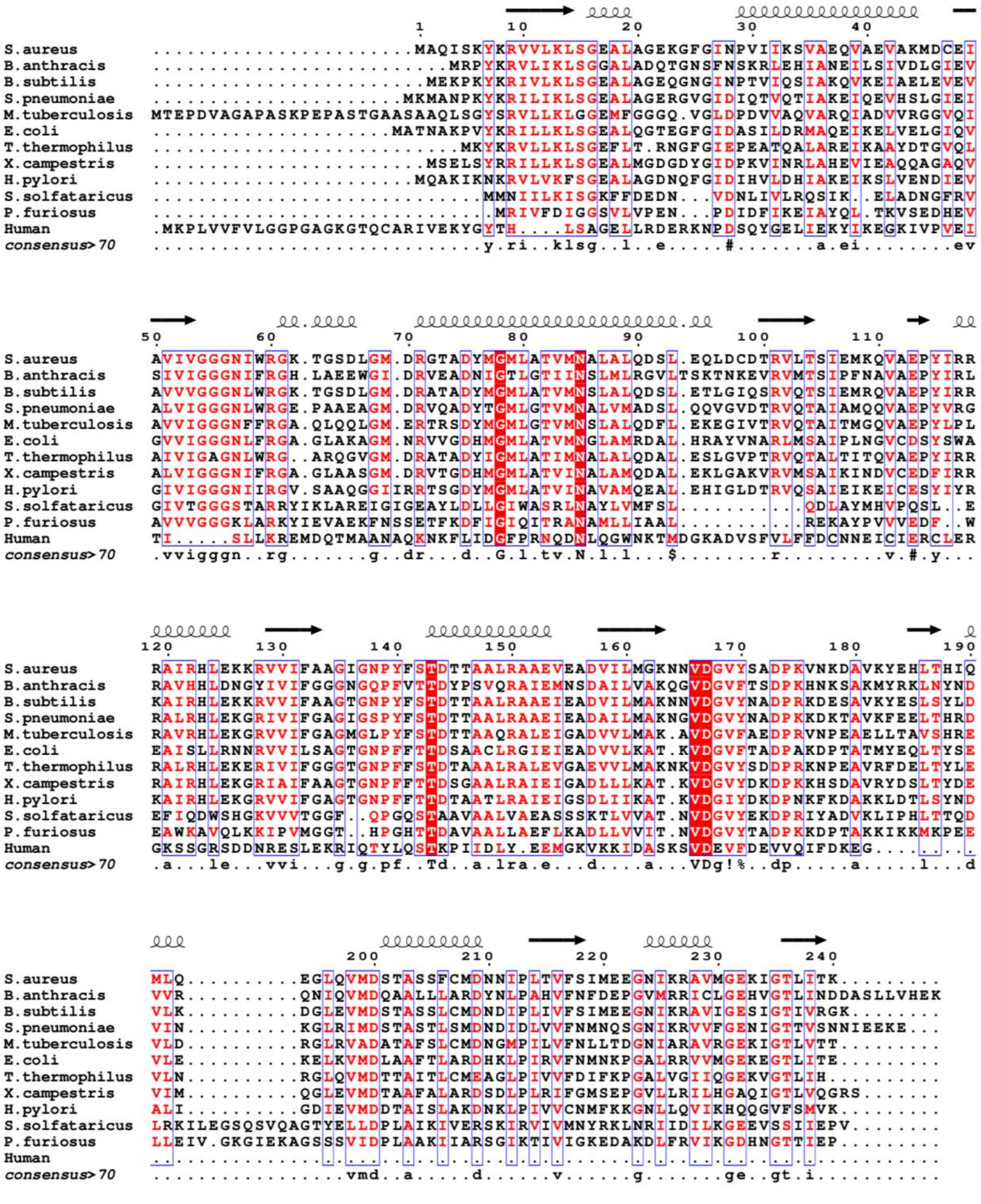

Supplementary Figure 4. Sequence alignment map of UMPK proteins across multiple species (ESPript 3.x / ENDscript 2.x). The species depicted are classified as follows: *S. aureus* (UniProt: Q2FZ22), *Mycobacterium tuberculosis* (UniProt: P9WHK5), *Bacillus anthracis* (UniProt: A0A6L7HKK4), *Bacillus subtilis* (UniProt: O31749), and *Streptococcus pneumoniae* (UniProt: Q8DQ50) are Gram-positive bacteria; *Escherichia coli* (UniProt: P0A7E9), *Helicobacter pylori* (UniProt: P56106), *Xanthomonas campestris* (UniProt: P59009), and *Thermus thermophilus* (UniProt: P43891) belong to Gram-negative bacteria; *Saccharolobus solfataricus* (UniProt: Q97ZE2)

and *Pyrococcus furiosus* (UniProt: Q8U122) are archaea. Notably, the human enzyme is a multifunctional UMP/CMP kinase (UniProt: P30085). Secondary structure assignments and residue numbering follow the *S. aureus* sequence. α-helices and β-sheets are indicated by spirals and long arrows, respectively. The consensus sequence (>70% identity) is shown below. Capital letters represent strictly conserved residues, while lowercase letters and symbols indicate relatively conserved or conservatively substituted residues.

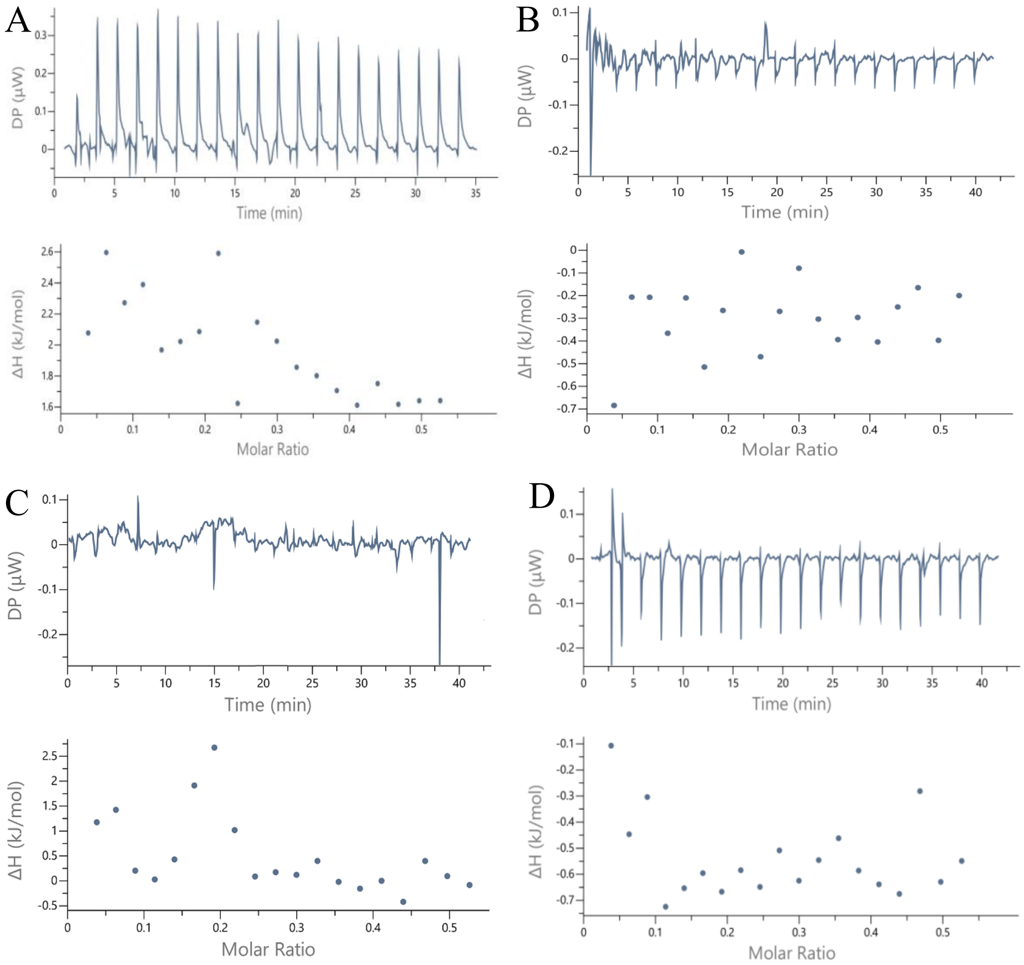

Supplementary Figure 5. Isothermal titration calorimetry (ITC) shows no measurable binding affinity between saUMPK and nucleoside monophosphates (NMPs). (A–D) Titration curves for 1 mM NMP ligands: (A) UMP, (B) AMP, (C) CMP, and (D) GMP, into 0.4 mM saUMPK.

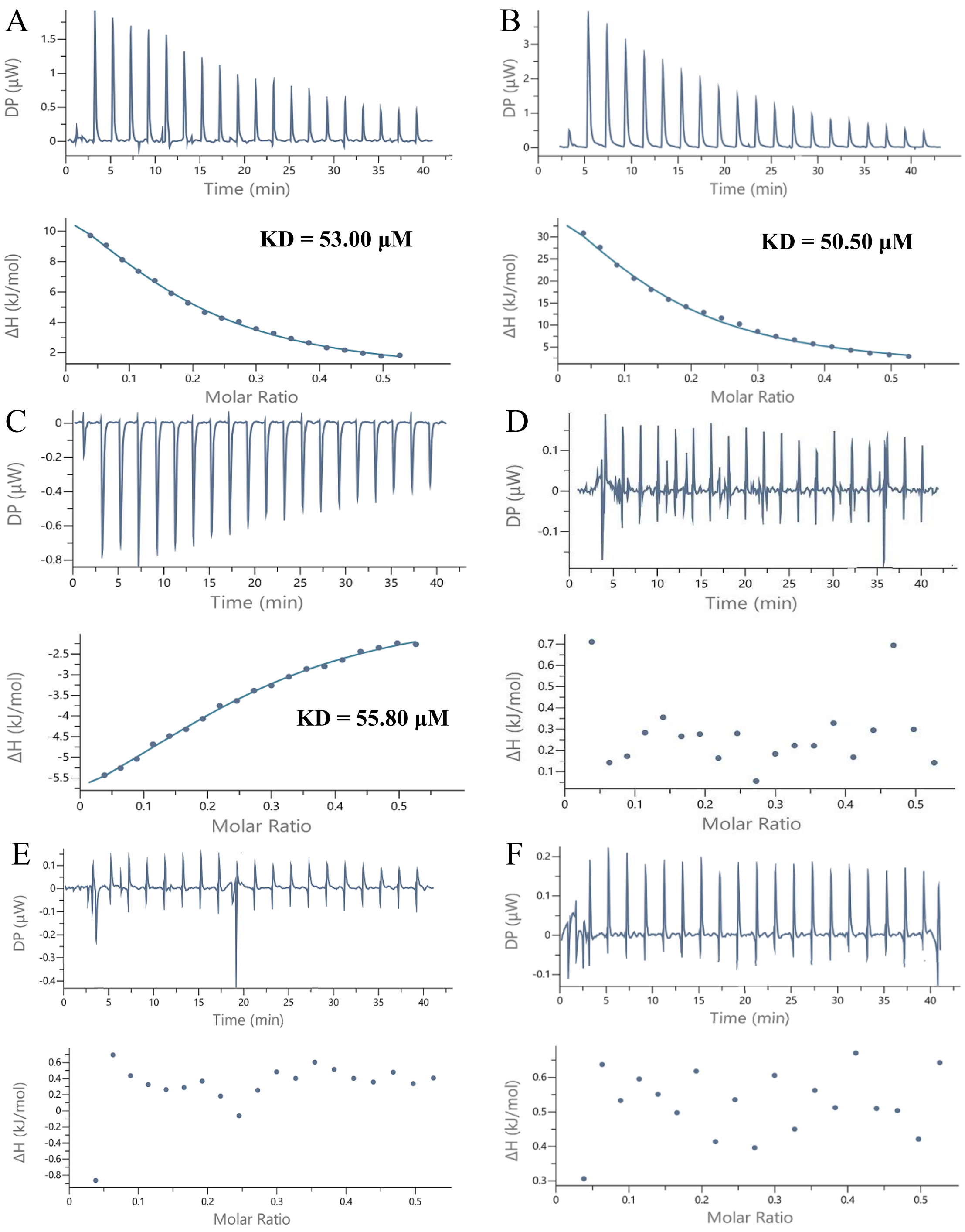

Supplementary Figure 6. Allosteric effector binding enables specific UMP recognition by saUMPK, as revealed by ITC. (A–C) Titration of 1 mM UMP into 0.4 mM saUMPK pre-bound to 1 mM allosteric effectors: (A) GTP, (B) ATP, and (C) UTP. (D–F) Titration of 1 mM of non-cognate NMPs into 0.4 mM saUMPK pre-bound to 1 mM GTP: (D) AMP, (E) CMP, and (F) GMP. UMP binding is observed only when allosteric sites are occupied by effector nucleotides (A–C).

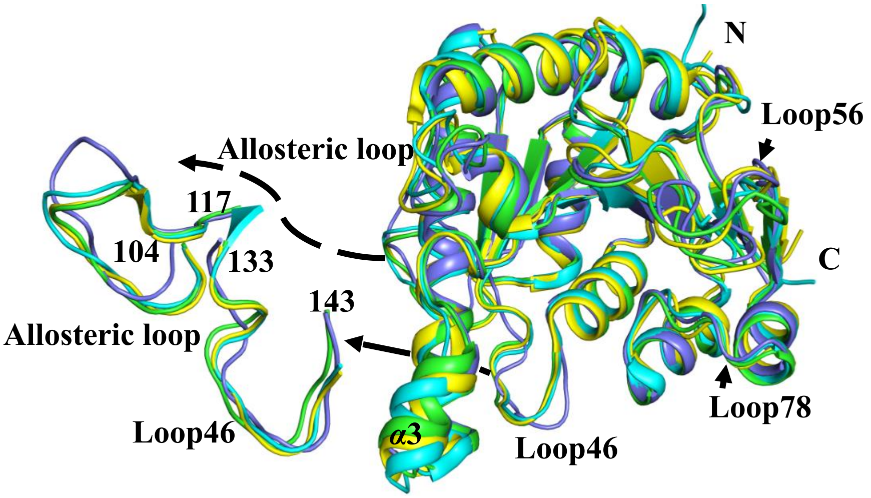

Supplementary Figure 7. Monomeric structural superposition of saUMPK-UMP with apo-form UMPKs from Gram-positive (*Streptococcus pyogenes*, PDB: 1Z9D; yellow) and Gram-negative (*Haemophilus influenzae*, PDB: 2A1F; blue) bacteria. Chain A (green) and chain B (slate) of the saUMPK-UMP complex are shown. In chain A, the allosteric loop and Loop46 adopt a bound-state conformation, consistent with those of *S. pyogenes* and *H. influenzae*. Chain B exhibits an apo-like conformation with significant conformational differences in both loops. An expanded view (left) highlights the dual-loop region in the right structure. Numbers at the loop termini indicate residue positions in saUMPK.

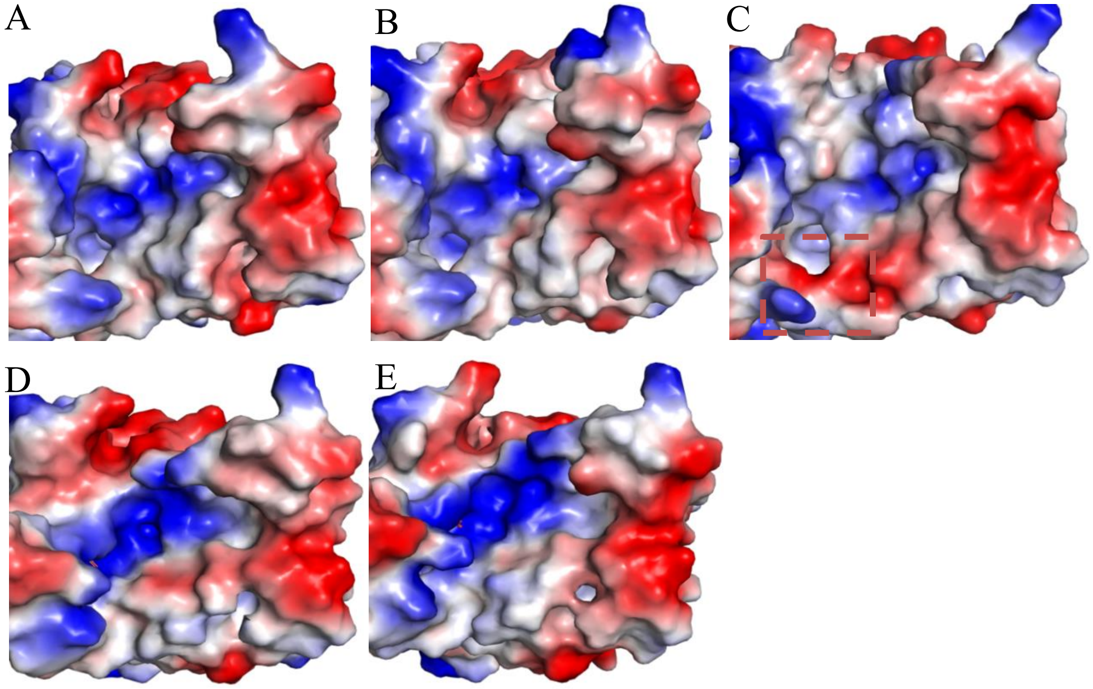

Supplementary Figure 8. Electrostatic potential maps of the active center in saUMPK monomers under different nucleotide-bound states (blue: positive charge; red: negative charge). (A)–(E) correspond to the saUMPK-GTP, saUMPK-UTP, saUMPK-UMP (apo-like), saUMPK-ATP/UMP, and saUMPK-UDP complexes, respectively. Allosteric loop and Loop46 are in a bound state (A–B, D–E) or a free state (C); the orange dashed area highlights the absent R71–D75–Y140 hydrogen-bond network. When nucleotides are bound at the allosteric site, the active center adopts an open conformation (A, B), but a closed conformation is observed in U-shaped hexamers (D, E).

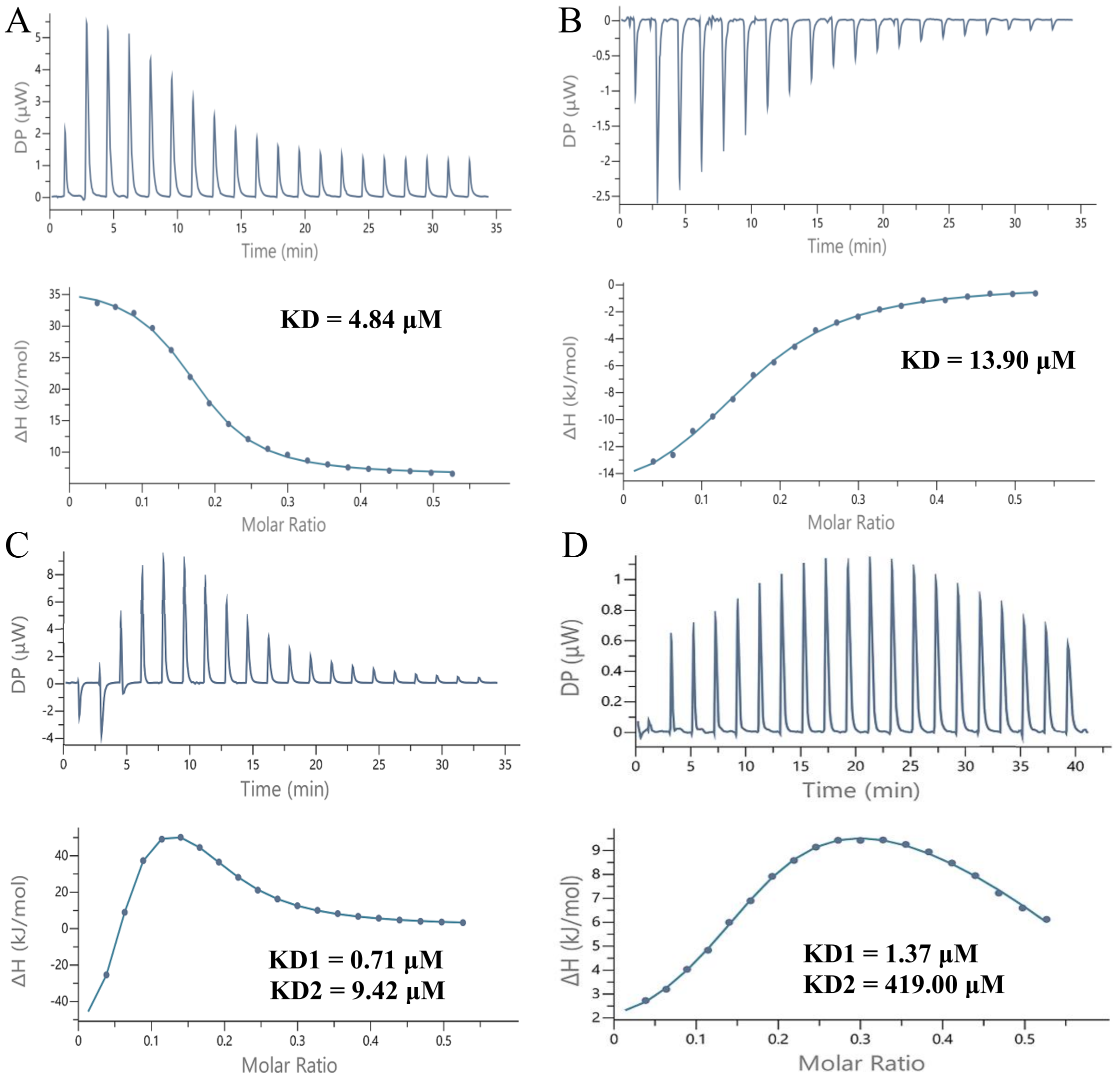

Supplementary Figure 9. Distinct nucleotide binding modes to saUMPK revealed by ITC. (A, B) Single-site binding to the allosteric site, as shown by the titration curves for UTP (A) and ATP (B). (C, D) Dual-site binding is demonstrated by the titration curves for GTP (C), targeting both the allosteric and ATP donor sites, and for UDP (D), targeting both the allosteric and UMP-binding sites. Protein concentration: 0.4 mM; nucleotide concentration: 1 mM.

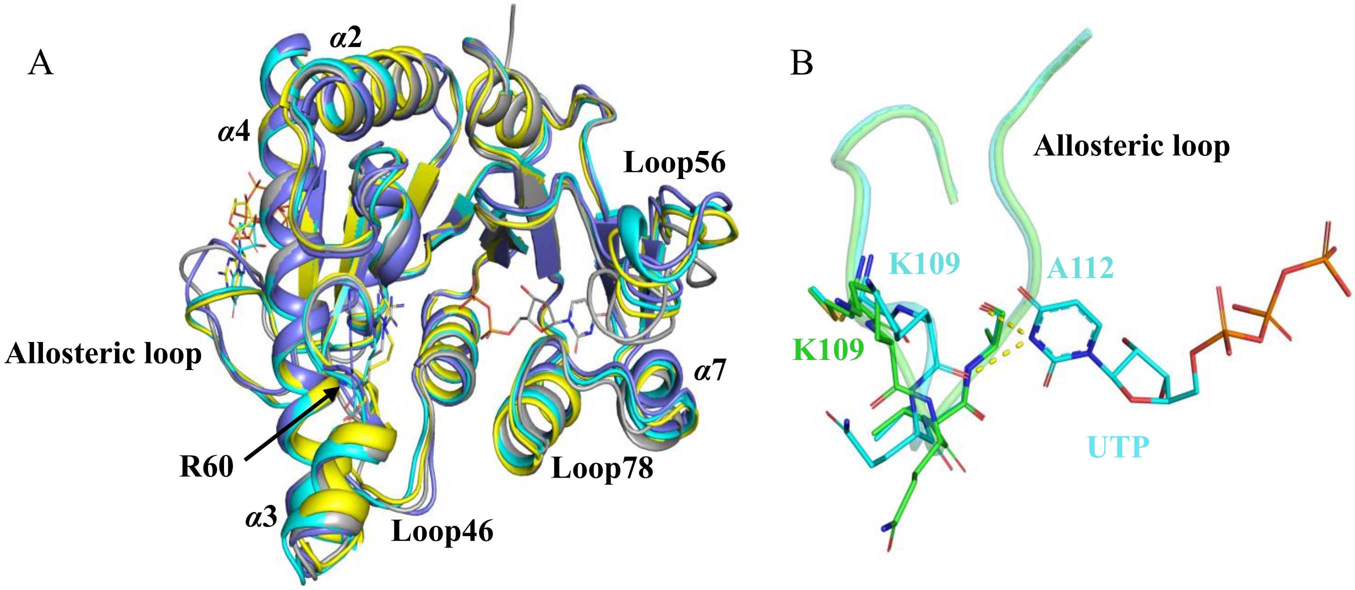

Supplementary Figure 10. Structural comparisons of saUMPK monomers and allosteric loop heterogeneity within the UTP-bound dimer. (A) Monomeric structure superposition of saUMPK-UTP (UTP-bound, blue; UDP-bound, concrete grey), saUMPK-GTP (GTP-bound, yellow), and saUMPK-UMP (apo-like reference, slate). C*α* RMSD = 0.46–0.71 Å. Significant effector-induced conformational differences include: the adoption of a bound state by the allosteric loop and Loop46, a subtle shift of R60 toward the UMP-binding site, an N-terminal shift of *α*2 toward the dimer interface, and a contraction of the ATP donor site (Loop56 and *α*7). UDP binding at the ATP donor site induces a more pronounced contraction of Loop56/*α*7. (B) Subtle differences in allosteric loops within the UTP-bound dimer. Both loops exhibit a bound state; however, the K109 side chain flips upon direct UTP binding (blue). This residue, along with A112, coordinates UTP via hydrogen bonds (yellow dashed lines). Nucleotides are shown as sticks (colored by chain).

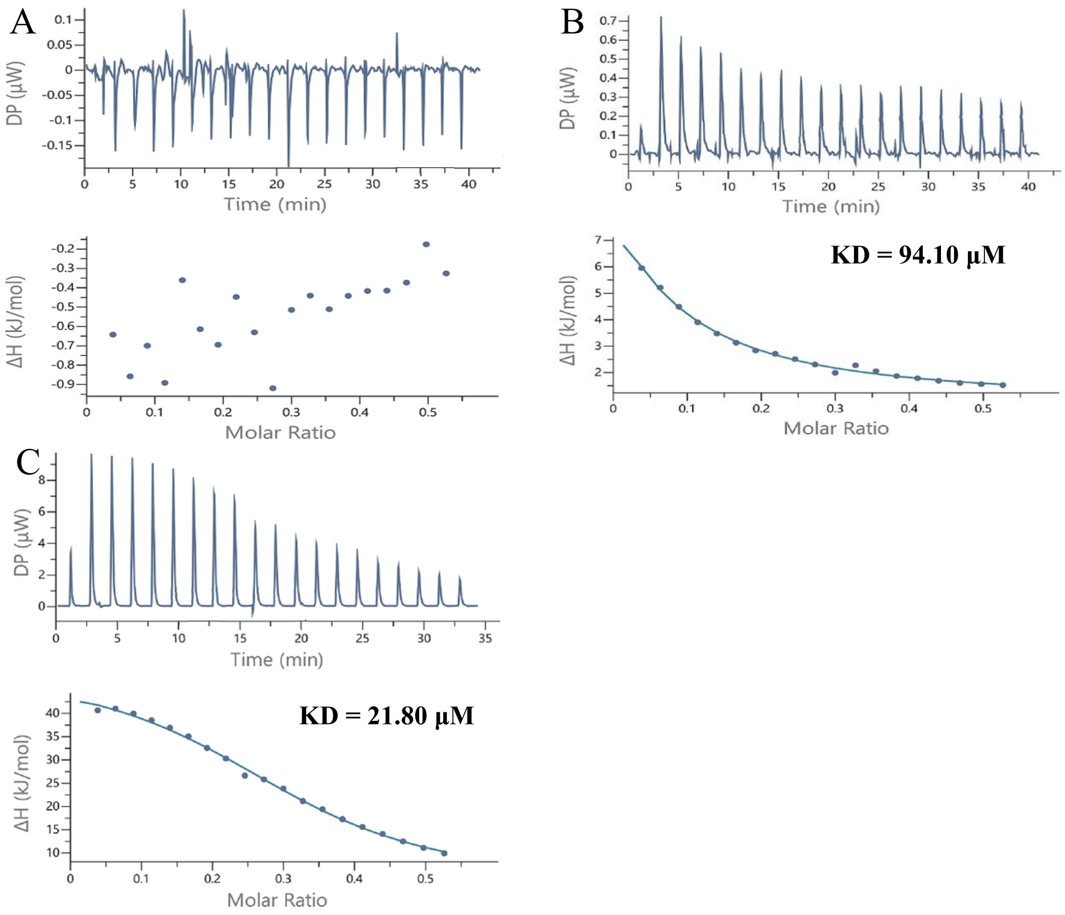

Supplementary Figure 11. Competitive nucleotide binding at the allosteric site via ITC. (A, B) Titration of 1 mM ATP into 0.4 mM saUMPK pre-bound to GTP at 1 mM (A) or 100 μM (B). (C) Titration of 1 mM GTP into 0.4 mM saUMPK pre-bound to 1 mM ATP. Binding affinity: GTP > ATP.

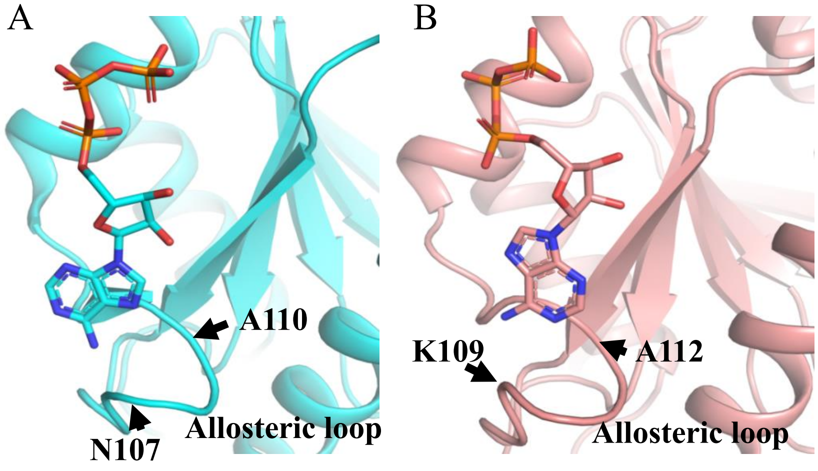

Supplementary Figure 12. Distinct ATP-binding conformations at the allosteric loops of UMPKs from *S. aureus* versus *B. anthracis*. (A) *B. anthracis* UMPK-ATP complex: the purine ring binds via N6 to N107 and A110. (B) *S. aureus* UMPK-ATP/GTP complex: the purine ring binds via N6 to K109 and A112. The orientation of ATP differs between the species, with the purine ring rotated approximately 180° in *S. aureus*. Nucleotides are shown as sticks.

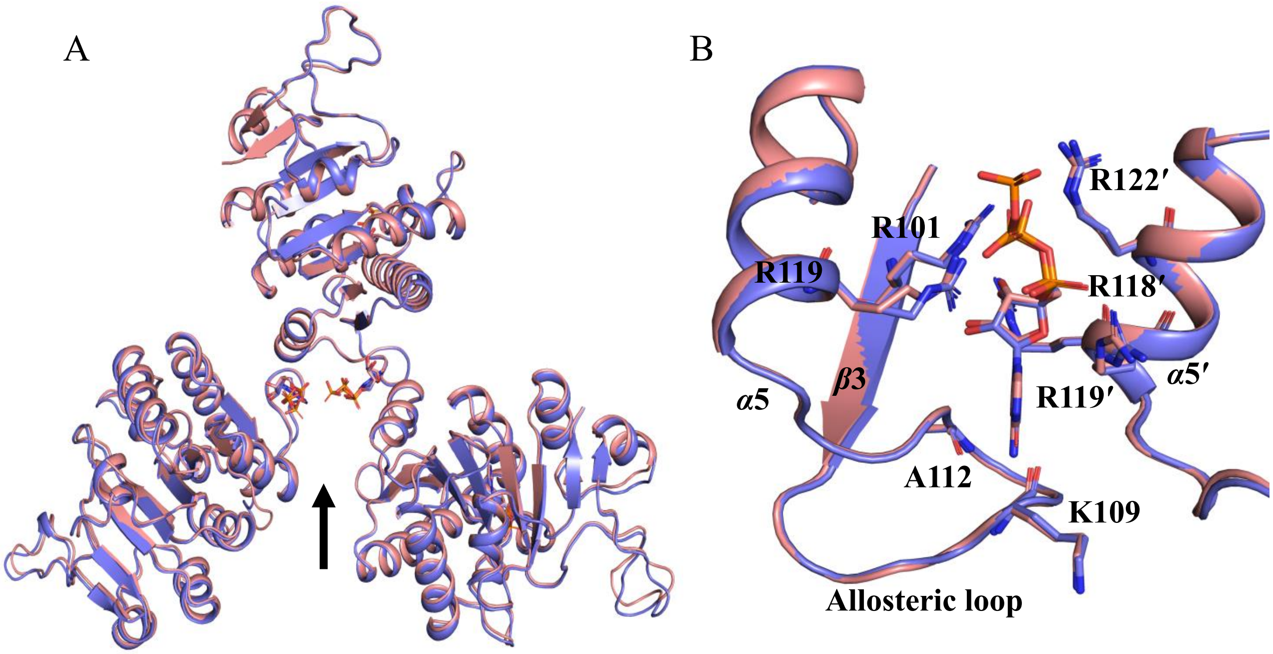

Supplementary Figure 13. Structural superposition of saUMPK-nucleotide binding complexes derived from U-shaped hexamers. (A) Superposition of trimer structures (composing a complete allosteric center; Cα RMSD = 0.33 Å) Each allosteric center binds only two ATP or UDP molecules at the allosteric sites; the remaining site (indicated by a black arrow) exhibits a ruptured conformation. (B) Structural superposition of the complete allosteric site, highlighting conserved hydrogen-bonding residues (labeled). Nucleotides are shown as sticks and colored by source complexes: pink (saUMPK-ATP/UMP) and slate (saUMPK-UDP).

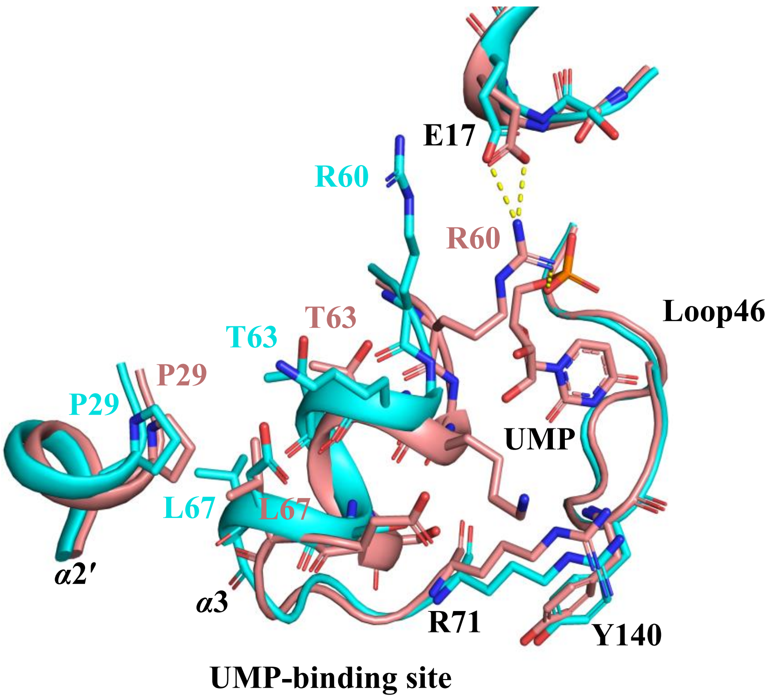

Supplementary Figure 14. Conformational changes near the UMP-binding site during the U-shaped transition, revealed by structural superposition. saUMPK-UTP and saUMPK-ATP/UMP (a U-shaped hexamer) are colored blue and pink, respectively. Key residues (P29, R60, T63, and L67) inherit the colors from their parent structures. In the U-shaped conformation, R60 forms hydrogen bonds with E17 and UMP (shown as a stick).

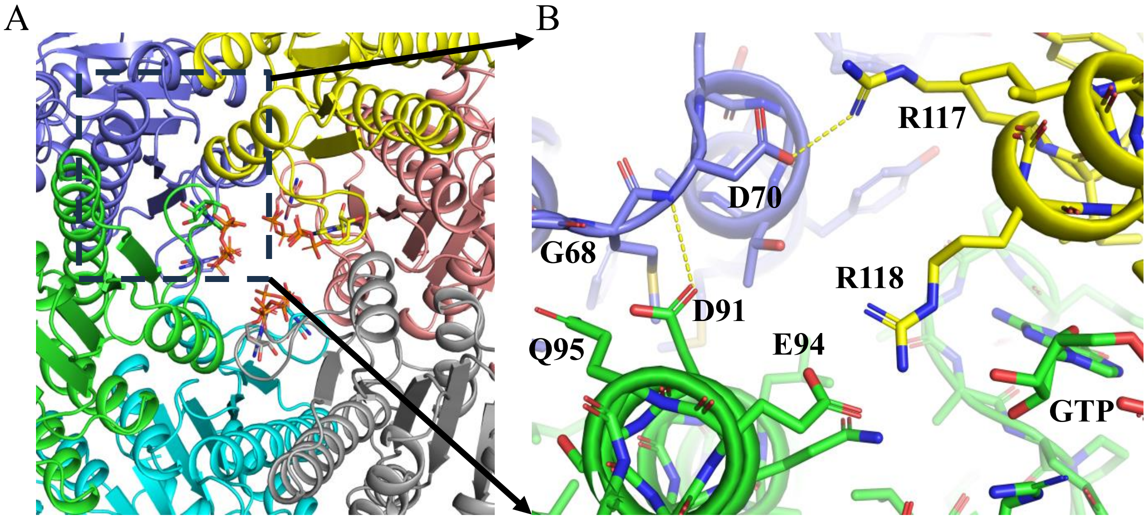

Supplementary Figure 15. Key polar interactions at the monomer interface in the saUMPK-GTP hexameric structure. (A) Central hexameric assembly. The black dashed box indicates one complete allosteric site. (B) Magnified view of the allosteric site (three adjacent monomers and bound GTP). D70 forms hydrogen bonds (yellow dashed lines) at both the standard dimer interface (green and slate) and a non-canonical interface defined by atypical subunit interactions (slate and yellow).

A B

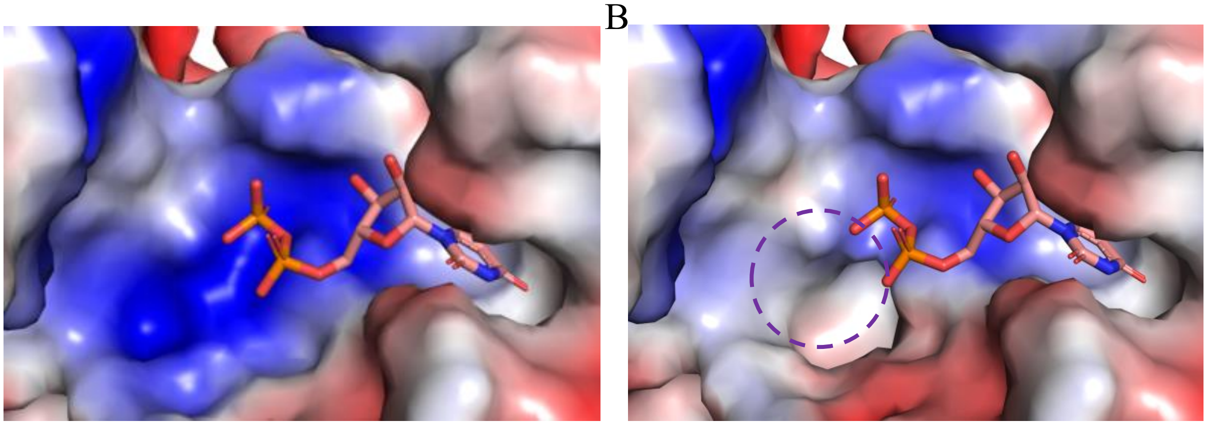

Supplementary Figure 16. Electrostatic potential comparison of the active center before and after the mutation in the saUMPK-UTP structure. Blue: positive; red: negative. (A) Wild-type (WT) active center (UDP-bound). (B) The K13A mutation (generated from A using PyMOL) shows an attenuated positive potential (purple dashed area). Nucleotides are shown as sticks.

1.2 Supplementary Tables

Supplementary Table 1. Primer sequences used in PCR reactions.

Primers	Primer sequences
WT_ Forward	5′-TCAC**CATATG**GCTCAAATTTCTAAATATAAAC-3′
WT_ Reverse	5′-GGTG**CTCGAG**TTATTTTGTAATTAACGTAC-3′
K13A_ Forward	5′-CAAATTTCTAAATATAAACGTGTAGTTTTG**GCA**CTAAGTGGTGAAGCGTTAGC-3′
K13A_ Reverse	5′-TTCTCCAGCTAACGCTTCACCACTTAG**TGC**CAAAACTACACGTTTATATT-3′
R60A_ Forward	5′-GTAATCGTTGGTGGCGGAAACATTTGG**GCA**GGTAAAACAGGTAGTGACTTAG-3′
R60A_ Reverse	5′-CATACCTAAGTCACTACCTGTTTTACC**TGC**CCAAATGTTTCCGCCACCAACG-3′
D70G_ Forward	5′-GGTAAAACAGGTAGTGACTTAGGTATG**GGC**CGTGGAACTGCTGATTAC-3′
D70G_ Reverse	5′-GCATACCCATGTAATCAGCAGTTCCACG**GCC**CATACCTAAGTCACTACC-3′
D75A_ Forward	5′-GACTTAGGTATGGACCGTGGAACTGCT**GCT**TACATGGGT-3′
D75A_ Reverse	5′-CATTCATTACAGTTGCAAGCATACCCATGTA**AGC**AGCAGTTCCAC-3′
M77N_ Forward	5′-TAGGTATGGACCGTGGAACTGCTGATTAC**AAT**GGTATGCTTGCAACTGTAAT-3′
M77N_ Reverse	5′-CTAAGGCATTCATTACAGTTGCAAGCATACC**ATT**GTAATCAGCAGTTCCACG-3′
M84N_ Forward	5′-GCTGATTACATGGGTATGCTTGCAACTGTA**AAC**AATGCCTTAGCATTACAAG-3′
M84N_ Reverse	5′-CTAAACTATCTTGTAATGCTAAGGCATT**GTT**TACAGTTGCAAGCATACC-3′
R101A_ Forward	5′-GATAGTTTAGAACAATTGGATTGTGATACA**GCA**GTATTAAC-3′
R101A_ Reverse	5′-CTTGCTTCATTTCAATAGATGTTAATAC**TGC**TGTATCACAAT-3′
E113A_ Forward	5′-TAACATCTATTGAAATGAAGCAAGTGGCT**GCA**CCTTATATTC-3′
E113A_ Reverse	5′-GTGTCTAATTGCACGACGACGAATATAAGG**TGC**AGCCACTTG-3′
R119A_ Forward	5′-GAAGCAAGTGGCTGAACCTTATATTCGTCGT**GCT**GCAATTAGAC-3′
R119A_ Reverse	5′-TAACTACGCGTTTCTTTTCTAAGTGTCTAATTGC**AGC**ACGACGAATATAAG-3′
R122A_ Forward	5′-GGCTGAACCTTATATTCGTCGTCGTGCAATT**GCA**CACTTAGAA-3′
R122A_ Reverse	5′-CAGCAAAAATAACTACGCGTTTCTTTTCTAAGTG**TGC**AATTGCACG-3′
G135A_ Forward	5′-GAAAAGAAACGCGTAGTTATTTTTGCTGCA**GCT**ATTGGAAACC-3′
G135A_ Reverse	5′-GCTGTAGTATCTGTAGAGAAGTATGGGTTTCCAAT**AGC**TGCAGCAAA-3′
N138A_ Forward	5′-GCGTAGTTATTTTTGCTGCAGGTATTGGA**GCC**CCATACTTC-3′
N138A_ Reverse	5′-GCCGCTGTAGTATCTGTAGAGAAGTATGG**GGC**TCCAATACC-3′
T143A_ Forward	5′-CTGCAGGTATTGGAAACCCATACTTCTCT**GCA**GATACTACAGCGGC-3′
T143A_ Reverse	5′-CTTCTGCAGCACGTAATGCCGCTGTAGTATC**TGC**AGAGAAGTATGGG-3′

The restriction sites and mutation regions are highlighted in bold.

Supplementary Table 2. X-ray diffraction and structure refinement statistics of six nucleotide-bound structures.

Data set	saUMPK-UTP	saUMPK-UDP	saUMPK-GTP	saUMPK-UMP	saUMPK-ATP/GTP	saUMPK-ATP/UMP
PDB ID	9UYX	9UVO	9UVK	9UVN	9UY1	9UVP
**Data collection**						
Space group	$P2_1$	$P4_12_12$	$P3_2$	$C2$	$P3_2$	$P4_12_12$
Unit-cell parameters						
a, b, c (Å)	76.94 114.79 101.34	73.34 73.34 301.41	194.26 194.26 116.78	117.36 96.30 75.84	194.79 194.79 118.75	72.55 72.55 298.06
α, β, γ (°)	90 90.63 90	90 90 90	90 90 120	90 103.45 90	90 90 120	90 90 90
No. reflections	77260 (4509)	22551 (3201)	110376 (5532)	12652 (2604)	114053 (5704)	25820 (1828)
R_{merge} (%)	11.10 (95.19)	11.5 (91.1)	14.4 (75.8)	15.0 (75.4)	15.0 (87.7)	9.18 (29.97)
$CC_{1/2}$ (%)	99.37 (77.39)	99.9 (89.7)	99.1 (68.8)	98.1 (66.2)	99.8 (85.4)	88.49 (71.90)
$I/\sigma I$	16.69 (1.67)	18.4 (3.7)	8.7 (1.6)	5.7 (1.4)	11.8 (4.1)	15.43 (1.90)
Completeness (%)	98.8 (98.1)	100.0 (100.0)	98.9 (100.0)	97.9 (98.3)	100.0 (100.0)	97.0 (97.0)
Multiplicity	6.40 (5.53)	13.8 (12.7)	4.1 (3.0)	4.2 (3.2)	7.3 (7.6)	5.18 (4.49)
**Refinement**						
Resolution (Å)	46.35–2.30 (2.38–2.30)	49.04–2.75 (2.85–2.75)	13.14–2.88 (2.98–2.88)	13.03–3.26 (3.38–3.26)	48.70–2.88 (2.98–2.88)	48.51–2.57 (2.66–2.57)
R_{work}/R_{free} (%)	19.98/24.20	21.06/25.62	20.31/25.70	23.60/27.63	20.29/25.78	21.37/24.50
B-factors (Å^2)	71.01	68.43	44.15	86.82	66.81	75.54

R.m.s.deviations						
Bond lengths (Å)	0.005	0.003	0.003	0.004	0.003	0.003
Bond angles (°)	0.684	0.572	0.515	0.616	0.595	0.593
Ramachandran plot (%) (favored/allowed/outliers）	98.4/1.6/0.0	97.9/2.1/0.0	98.0/2.0/0.0	97.7/2.3/0.0	97.8/2.2/0.0	97.9/2.1/0.0

$R_{merge}=\sum_{hkl}\sum_i|I_i(hkl)-\langle I(hkl)\rangle|/\sum_{hkl}\sum_i I_i(hkl)$, where Ii(hkl) indicates the ith measurement of the observed diffraction intensity at a given hkl direction, <I(hkl)> indicates the arithmetic mean of the observed diffraction intensities at a given hkl direction. The values in parentheses indicates the statistics data for the highest resolution shell.

Supplementary Table 3. Structural composition of saUMPK complexes in an asymmetric unit.

**Complex**	**No. of monomers**	**No. of allosteric centers**	**Nucleotide occupancy**
saUMPK-UTP	6 (1 hexamer)	2	2 UTP (1 per allosteric center) at allosteric sites; 3 UDP at ATP donor sites
saUMPK-UDP	3	1	2 UDP at allosteric sites; 2 UDP at UMP-binding sites
saUMPK-GTP	18 (3 hexamers)	6	GTP occupies all allosteric sites
saUMPK-UMP	3	1	1 UMP at UMP-binding site
saUMPK-ATP/GTP	18 (3 hexamers)	6	GTP and ATP (ADP) occupy all allosteric sites
saUMPK-ATP/UMP	3	1	2 ATP at allosteric sites; 2 UMP at UMP-binding sites

Each allosteric center is formed by three saUMPK monomers, with their allosteric sites converging toward the center axis. These two trimeric subunits assemble into a complete hexameric architecture through staggered stacking along the vertical axis. Complexes lacking the “hexamer” label in the “No. of monomers” column contain a single trimer-like assembly.
